# School-based strategies to increase physical activity and reduce sedentary behaviour in students with disability: protocol of the TransformUs All Abilities hybrid type II implementation-effectiveness trial

**DOI:** 10.1136/bmjopen-2025-105311

**Published:** 2025-11-09

**Authors:** Emiliano Mazzoli, Ana María Contardo Ayala,, Harriet Koorts, Anna Timperio, Natalie Lander, David Revalds Lubans, Nicola D Ridgers, Kate Louise McCann Anderson, John Cairney, Lisa Michele Barnett, Jo Salmon

**Affiliations:** 1 Institute for Physical Activity and Nutrition (IPAN), School of Exercise and Nutrition Sciences, Faculty of Health, Deakin University, Geelong, Victoria, Australia; 2 School of Health and Social Development, Faculty of Health, Deakin University, Geelong, Victoria, Australia; 3 Global Sport and Movement Collaborative, College of Human and Social Futures, University of Newcastle, Callaghan, New South Wales, Australia; 4 The University of Newcastle Hunter Medical Research Institute, New Lambton Heights, New South Wales, Australia; 5 Alliance for Research in Exercise, Nutrition and Activity, University of South Australia, Adelaide, South Australia, Australia; 6 School of Computing Technologies, STEM College, RMIT University, Melbourne, Victoria, Australia; 7 School of Human Movement and Nutrition Sciences, The University of Queensland, Saint Lucia, Queensland, Australia

**Keywords:** Disabled Persons, Child, Adolescent, Pragmatic Clinical Trial, Schools

## Abstract

**Introduction:**

*TransformUs* is a multicomponent school-based programme that offers teachers professional learning and resources aligned with the Australian curriculum to promote physically active teaching and learning, a supportive environment and physical activity opportunities during recess and lunch. The programme was originally developed for students in mainstream primary schools and has been proven efficacious for increasing physical activity and reducing sedentary behaviour in children without disability. The programme has been adapted for delivery with students with disabilities in primary and secondary schools (*TransformUs All Abilities*). This project aims to determine the implementation at scale and effectiveness of the *TransformUs All Abilities* programme to increase physical activity among primary and secondary school children and adolescents with disability. This protocol describes the hybrid implementation-effectiveness trial that will be used for this evaluation.

**Methods and analysis:**

This study employs a hybrid type II implementation-effectiveness trial to evaluate the *TransformUs All Abilities* programme, targeting all government and independent, primary and secondary schools in Victoria, as well as special and mainstream secondary schools in Queensland and South Australia (n=2173 eligible schools). The effectiveness trial will focus on a subgroup of government/independent special schools for students with mild to moderate intellectual disability in Victoria, involving up to three intervention and three waitlist control schools (n=61 eligible schools). In both trials, outcomes will be guided by the RE-AIM framework focusing on reach, adoption and implementation (implementation trial) and effectiveness (effectiveness trial), with data collected at baseline and 6 months. The effectiveness trial will focus on students’ device-measured physical activity and sedentary behaviour—primary outcomes—and sleep, physical literacy and cognitive functions—secondary outcomes. Teacher feedback on the programme’s adaptation and their experience with programme implementation will also be collected, alongside qualitative feedback from a subsample of students regarding engagement/enjoyment and suggestions for improvements. Implementation data will be analysed descriptively and using linear mixed models to test changes over time. Effectiveness outcomes will be analysed using linear mixed models to compare intervention and waitlist control, accounting for confounding and school/classroom clustering. Interview data will be thematically analysed.

**Ethics and dissemination:**

Ethical approval for this trial was obtained from the Deakin University Human Research Ethics Committee (2021-368). Clearance to conduct research in schools was also obtained from the Education Departments of Victoria (2023-004726), Queensland (550/27/2592) and South Australia (2022-0020). Informed consent is required for participation in the study. School staff can enrol in the implementation trial via the *TransformUs* website, while the effectiveness trial requires organisational, staff, parental/carer consent and student assent. Results will be disseminated through academic publications, scientific conference presentations and summary reports to schools, parents and partner organisations.

**Trial registration:**

ACTRN12622001082796; Universal Trial Number: U1111-1281-1103; ACTRN12622001050741: U1111-1280-8828.

**STRENGTHS AND LIMITATIONS OF THIS STUDY**

- *TransformUs All Abilities* builds on the proven success of the original *TransformUs* programme, by providing adaptations to the programme using a strengths-based approach inspired by principles of inclusive education and guided by the TREE model (Teaching style, Rules, Equipment and Environment), which offers a practical framework for modifying activities to support participation and engagement of all children.
- *TransformUs All Abilities* leverages *TransformUs’* strong theoretical foundations and alignment with the Australian curriculum, as well as government targets and priorities, making it a promising initiative for adapting and extending evidence-based strategies to enhance physical activity and reduce sedentary behaviour among students with disability, an underserved population.
- The hybrid implementation-effectiveness trial and the use of mixed methods allow for the synchronised collection of data on implementation and effectiveness outcomes of an intervention in a real-world setting, contributing to the evidence base for at-scale implementation of initiatives designed to promote the inclusion of children and adolescents with disability; with online resources and supporting materials provided free of charge during both trials, the programme is readily accessible to all school staff, facilitating adoption and potential scalability across Australia.
- The delivery relies on the active participation, commitment and skills of school staff which may vary significantly across different schools, and schools may differ in their capacity, resources and approach to implementing the intervention; comprehensive support strategies are offered—including resources, professional development sessions, webinars and ongoing support from research staff—to address potential biases arising from differences in intervention delivery across schools.
- The trial’s 6-month duration provides initial insights into programme implementation and short-term outcomes, though it may limit understanding of real-world impact, and while implementation includes all school types and has the potential to benefit students with a wide range of (dis-)abilities, effectiveness mainly reflects outcomes for students with intellectual disability and may not be generalisable to other disability types.

## Introduction

 Regular physical activity is critical for children’s healthy development. It can help improve strength, coordination, balance and flexibility,^1^ boost cognitive functions^2 3^ and enhance mood and self-esteem,^4^ all essential elements for independent living later in life. These benefits also apply to children and adolescents with disabilities,^5^,^7^ which can include physical, sensory, intellectual and developmental disabilities. Approximately one in 10 children and adolescents worldwide (nearly 240 million) have a disability and regularly experience discrimination and stigma, leading to systematic exclusion from societal activities, and greater disadvantage and health-related risks compared with peers without disability.^8^ Based on the available evidence of benefits/risks,^9^ the World Health Organization recommends that children and adolescents should engage in an average of at least 60 min of moderate- to vigorous-intensity physical activity (MVPA) each day, incorporate vigorous-intensity aerobic and muscle and bone strengthening activities at least 3 days a week and reduce the time spent being sedentary.^10^ Importantly, recent guidelines explicitly emphasise this target for children and adolescents with disability, as well as those without disability.^11^

Sedentary behaviour—defined as behaviours characterised by an energy expenditure ≤1.5 metabolic equivalents while in a sitting, lying or reclining posture^12^—is also a health-related risk factor, independent of physical activity. Evidence of associations between sedentary behaviour and negative health outcomes is more consistent in adults^13 14^ than in youth,^15^ with negative associations in health outcomes found for children’s recreational screen time and inconclusive associations found for device-measured sitting time. While some studies have explored sedentary behaviour in children and adolescents with disability,^16^ little attention has been paid to the health-related impact of this behaviour or to interventions for addressing this public health issue in this population.

Children and adolescents without disability are insufficiently active across the world,^17^ and this may translate to poorer health and reduced quality of life in adulthood. Globally, fewer children with disabilities meet physical activity guidelines (ie, 20%–26%),^18^ compared with those without disabilities (27%–33%).^17^ The low physical activity levels of young people with disabilities are also confirmed by studies that assessed physical activity with accelerometers.^19^,^21^ In addition, children with disabilities have lower levels of well-being,^8^ fitness^22^ and motor skills^6^ and are more likely to develop obesity and chronic conditions.^8 23^ Lack of physical and social skills, social support and appropriate programmes/facilities are often reported as barriers to participation.^24 25^ It is crucial that all children and adolescents, especially those with a disability, have access to daily physical activity opportunities that are tailored to their needs.

Most of the interventions aimed at providing opportunities for physical activity for children with disabilities are conducted in clinical settings^26^ and there are several limitations related to the scientific rigour and scalability of the interventions conducted so far.^27^ Conducting ‘real-world’ physical activity and sedentary behaviour interventions has been identified as a critical public health priority.^28^ Children spend a great part of their waking hours in school, a setting in which children with disabilities tend to be more active compared with out-of-school hours.^29^ Whole-of-school approaches to physical activity incorporate curriculum-based and non-curriculum-based strategies (eg, supportive environments) to help students move more and sit less during the school day. These include providing quality physical education, encouraging active travel to and from school, offering before-school and after-school physical activity programmes, providing physical activity opportunities during recess and lunch, embedding activity in the classroom (ie, active lessons and active breaks) and ensuring that the offered activities are inclusive and accessible for students with disabilities.^30^ Research shows that these approaches are feasible and effective for implementation with children without disability,^31^,^33^ but there is limited understanding of the feasibility, effectiveness and implementation of whole-of-school physical activity programmes on children and adolescents with disability.

Some evidence of the feasibility of school-based physical activity approaches in children and adolescents with disability has recently emerged,^34 35^ although very few school-based interventions for children and adolescents with disability aimed to increase physical activity or reduce sedentary behaviour.^36 37^ Rather, most programmes have focused on clinical and rehabilitative outcomes.^38^ Moreover, very few interventions have been delivered by teachers or school staff (ie, teacher aides or other support staff),^37^ with the majority being delivered by researchers or therapists, which limits the scalability of effective interventions.^39^ Furthermore, many interventions have mostly been tested in just one or two schools,^36 39 40^ which limits the generalisability of such strategies. Further research is needed to determine whether school-based physical activity programmes for children and adolescents with disability can become an integral part of school practice.

*TransformUs*^41^ is an efficacious whole-of-school programme delivered in the primary school setting that improved health, successfully reduced sitting and increased physical activity at school and home in children without disability.^42^,^45^ The programme consists of the following intervention components: (1) health lessons; (2) active lessons (eg, physically active maths lessons); (3) active breaks (eg, breaking up class sitting with short physical activity bouts in the classroom); (4) active homework; (5) environmental modifications to the classroom and school environments to facilitate physical activity and (6) parent newsletters. The programme also includes professional learning resources for teachers and other relevant resources for school leaders designed to foster a whole-of-school culture towards physical activity. The implementation of *TransformUs* has been tested in children without disability^46 47^ and the programme is currently being implemented ‘at scale’ across Victoria (Australia) in collaboration with 16 key organisations (eg, State of Victoria Department of Education and Training).^48^ Although such strategies are effective in primary school children without disability,^43^ there is currently minimal evidence on what types of strategies are best suited for children and adolescents with disability and/or how to best tailor such strategies to different needs.^49^ Recognising this gap, and in response to direct demand from educators and schools serving students with disability, the programme has recently been adapted (ie, *TransformUs All Abilities*) to ensure that the additional needs of primary and secondary students with disability, in mainstream or special schools, are considered.

This article describes the adaptation of *TransformUs* and the hybrid implementation-effectiveness trials that will evaluate *TransformUs All Abilities* in real-world settings. The overarching aim of this project is to determine the implementation at scale and effectiveness of the *TransformUs All Abilities* programme to increase physical activity among primary and secondary school children and adolescents with disability. Specific research objectives are as follows: (1) to determine the reach (eg, number and characteristics of *TransformUs* users who indicate having students with disability in their classroom), adoption (eg, reasons for uptake of the programme), implementation (eg, barriers and facilitators to implementing the programme) and maintenance (eg, teacher views of factors affecting the long-term sustainability) of *TransformUs All Abilities* in schools (ie, implementation trial); and (2) to test the effectiveness of *TransformUs All Abilities* on physical activity, sedentary time, physical literacy and cognitive functions in students with disability (ie, effectiveness trial).

## Methods and analysis

This protocol is reported in accordance with the Standard Protocol Items: Recommendations for Interventional Trials (SPIRIT) checklist.^50^

### Project overview

*TransformUs All Abilities* is an adapted version of the original *TransformUs* programme.^41^ The adaptation process was informed by a review of the literature on school-delivered physical activity interventions in children and adolescents with disability. From the literature, we identified only a few teacher-implemented interventions targeting students with disabilities, and none that used a whole-of-school approach. This represents a potential challenge to scaling up interventions, even those found to be effective, as their implementation may require significant adaptation to real-world school settings.

The adaptation of *TransformUs* followed a strengths-based approach inspired by principles of inclusive education. Targeting a wide range of disabilities, grouping them appropriately was an initial challenge, as people with disabilities may present highly heterogeneous physical, cognitive, sensory and behavioural needs. While frameworks like the International Classification of Functioning, Disability and Health (ICF)^51^ and the Diagnostic and Statistical Manual of Mental Disorders^52^ offer detailed categorisations, these are complex and not practical for group-based interventions. In designing *TransformUs All Abilities*, we adopted a broad and inclusive classification of disability aligned with the social model of disability, which emphasises the role of societal and environmental barriers in limiting participation, rather than focusing solely on individual impairments. Therefore, we adopted a broader approach that acknowledges the diversity of experiences and needs among children and adolescents with disabilities. Rather than relying on narrow diagnostic categories, we aligned our classification with the Nationally Consistent Collection of Data on School Students with Disability,^53^ which identifies four broad categories of disability: sensory, physical, cognitive and social–emotional. This framework reflects the social model of disability, recognising that difficulties within each of these broad domains often arise from the interaction between individuals and their environments, rather than from impairments alone. By framing disability in this way, *TransformUs All Abilities* aims to promote universal design principles and inclusive practices that support participation for all students, regardless of diagnosis or classification. This simple and universal framework focuses on students’ functional needs and provides strategies applicable to all students, not just those with disabilities.

*TransformUs All Abilities* incorporates the TREE model to support teachers in implementing inclusive practices.^54^ TREE stands for Teaching style (how activities are presented), Rules (modifications to the activity structure), Equipment (tools to enhance participation) and Environment (adjustments to space, sound and interactions). The *TransformUs* website offers professional development resources that explain disability and inclusion, provide an overview of the TREE model and demonstrate its use in adapting teaching materials for students with disabilities.

Adaptation suggestions are tailored to each of the four disability categories, emphasising students’ abilities and enjoyment rather than their limitations. The adaptations are designed to support students with a wide range of functional and cognitive abilities, aiming to enhance enjoyment and engagement and ensure the programme is both accessible and meaningful for all students. These suggestions are available in the professional development section of the website and are integrated into 65 existing *TransformUs* resources: 18 active breaks and 47 active lessons (16 English, 16 Maths and 15 Science). Examples of adapted resources are provided in online supplemental file 1.

### Patient and public involvement

Patients and the public were involved throughout the research process using a participatory approach. Initial involvement occurred during the formative phase, where we conducted interviews with 10 parents of children with disabilities and representatives from nine key organisations (eg, professional organisations in disability and/or education; under review). Findings from these interviews guided the design of the intervention, helped refine the research questions and helped identify challenges and priorities relating to using physically active strategies within the school setting with children and adolescents with a disability. Additionally, we engaged through iterative codesign workshops with teachers, school leaders and professionals in education and disability. This helped shape the intervention materials, while ensuring these materials are feasible to use and acceptable in school settings.

The results from the participatory research highlighted that *TransformUs* resources and equipment needed to be tailored to meet the specific needs of all students to be successfully implemented among students with disabilities. These adaptations had to be easy for teachers to use, align with children’s perceptions and interests in physical activity and support a shift in school culture towards prioritising physical activity. These inputs were critical in simplifying intervention components and aligning them with school routines.

Results will be shared with schools, parents, disability organisations and the scientific community through tailored summaries, presentations and accessible formats.

### Study design

A hybrid type II implementation-effectiveness trial design^55^ has been adopted to enable synchronous collection of data on intervention implementation and effectiveness in a real-world setting.^56^ The programme will be offered nationally through the *TransformUs* website, but data will only be collected in three states. The implementation trial is being offered to school leaders and teachers working in government and independent special schools in Victoria, Queensland and South Australia (208 special schools). It will also be offered to those working in a government or independent mainstream primary or combined school (1175 primary schools and 237 combined schools in Victoria) or secondary schools (553 schools in Victoria, Queensland and South Australia), if on registration to the *TransformUs* website, they indicate that they have students with additional needs in their class. Victorian primary schools and teachers who have already registered for the *TransformUs* programme (714 schools and 2088 teachers, as of August 2024) will also be notified of the availability of these new resources and invited to participate in the evaluation.

**Figure 1:**
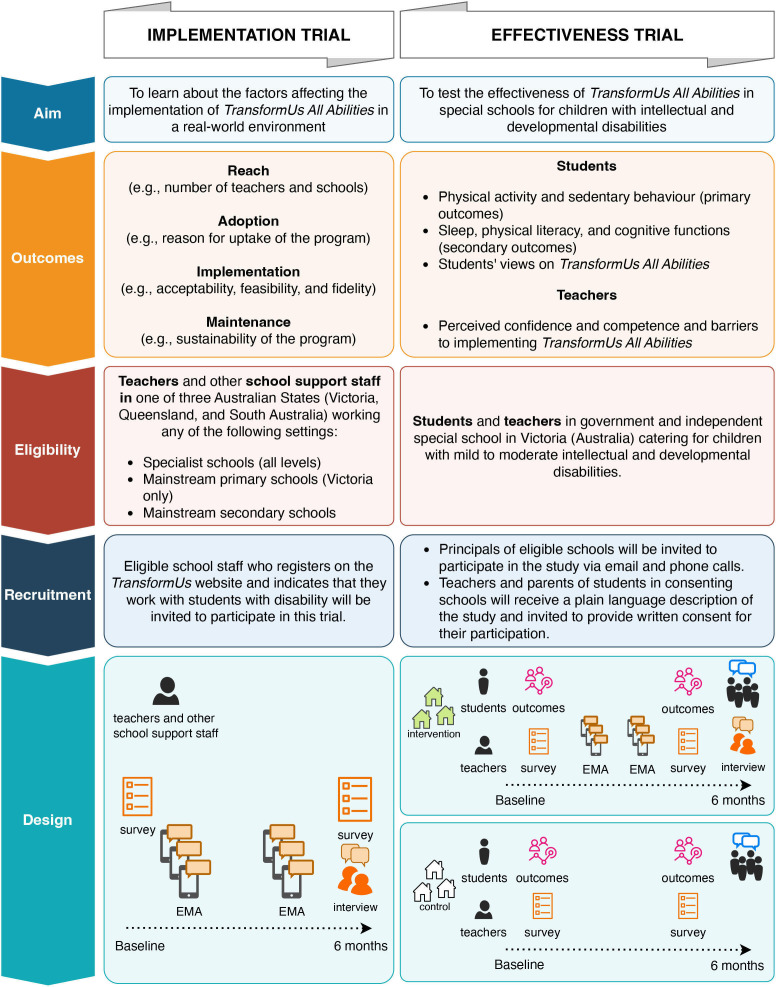
*TransformUs All Abilities* implementation-effectiveness trial overview. EMA, Ecological Momentary Assessment.

The parallel effectiveness trial component will involve up to six government special schools for students with mild to moderate intellectual disability in Victoria (Australia) (three intervention and three waitlist control schools from 61 eligible schools). Schools and school staff involved in the effectiveness trial are excluded from the implementation trial. Support strategies for teachers and schools to incorporate *TransformUs All Abilities* in their practice include the *TransformUs* website/resources, professional development sessions (in-person for the effectiveness trial and online for the implementation trial), text reminders and support from the research staff. The research process is depicted in figure 1. More detail is provided in the measures section.

### Procedures and participants

#### Implementation trial

The adapted programme will be made available nationwide through the *TransformUs* website. All school staff in Australia will be able to register and access the resources. Due to funding constraints and practical implications with obtaining ethical approval from the Departments of Education of each Australian State and Territory, only mainstream and specialist schools located in Victoria, Queensland and South Australia will be invited to provide implementation data. School staff will be required to sign up via the *TransformUs* website (https://transformus.com.au/) to access the programme. On registration, if they indicate that they have children with disabilities in their class, they will be invited to take part in the evaluation. Teachers working in Victorian schools who are already registered for the original *TransformUs* programme (for mainstream primary schools; ethics approval HEAG-H 28-2017) will be notified of new *TransformUs All Abilities* resources available on the website and invited to participate in the evaluation. Surveys will be conducted online via Qualtrics or Research Electronic Data Capture (REDCap). Interviews will be conducted via phone or online (Zoom or Teams). Ecological Momentary Assessment (EMA) to determine implementation of programme components will be conducted via text message.

The focus of this trial will be on investigating the reach (eg, number of teachers and schools), adoption (eg, number of accesses to resources and professional development material) and implementation-level outcomes (eg, acceptability, feasibility, fidelity, penetration, sustainability, satisfaction)—as defined by Proctor *et al*^57^—of the *TransformUs All Abilities* strategies by teachers in schools.

Estimating the exact percentage of school staff potentially registering for this initiative is challenging given the integrated nature of the *TransformUs All Abilities* programme within the *TransformUs* website (also including primary and secondary). The invitation for the implementation trial will only be made to school staff who indicate that they have children with disabilities in their class. According to the 2023 data from the Australian Bureau of Statistics,^58^ there were 9629 government/independent primary, secondary, combined and special schools in Australia. About 56% of the 3877 government/independent schools in the three States we intend to target for the implementation trial are potentially eligible to enrol in the trial (ie, n=2173 schools, including 208 special and 1965 mainstream schools). In 2023, there was a total of 167 107 teaching or specialist support school staff working in any government/independent schools in Victoria, Queensland and South Australia. Although the Australian Bureau of Statistics does not offer a breakdown of staff by school type,^58^ considering that the eligible schools represent 56% of the total number of schools in these States, the likely total number of staff eligible to enrol in the trial should not exceed 93 661 staff.

#### Effectiveness trial

The effectiveness trial will be run in Victoria for practical reasons (ie, funding constraints regarding in-school data collection and professional learning sessions, and multiple ethics approvals that would be required from Departments of Education in different states/territories). Up to six Victorian special schools (up to n=3 intervention and n=3 waitlist control; out of n=61 eligible schools) will be recruited for the effectiveness trial. School staff in the intervention school will be offered at least one in-person professional development opportunity at their school. School staff will also access the *TransformUs All Abilities* training material, programme components and programme resources through the *TransformUs* website (https://transformus.com.au) and will be encouraged to use the strategies in their classrooms/school.

Intervention effectiveness will be assessed in relation to device-measured students’ physical activity and sedentary time (primary outcomes), sleep, physical literacy and cognitive functions (secondary outcomes), as well as teachers’ confidence and competence in implementing the *Transform All Abilities* programme (secondary outcomes). Teachers’ feedback on their experience of delivering the programme and the perceived barriers and facilitators to implementation will be sought with audio-recorded interviews/focus groups at the end of the trial. Additionally, at the end of the trial, we will conduct a 45 min focus group session with a small group of students at each intervention school to understand how students with disabilities view the opportunities for physical activity throughout the school day. With these discussions, we will aim to understand (1) students’ perceptions of the *TransformUs All Abilities* programme; (2) their enjoyment in relation to the programme and (3) perceived barriers and facilitators to the implementation of physical activity strategies in their schools.

The principal/nominated school leader will help to oversee the implementation of the intervention in the school. A nominated school contact/liaison will distribute information on the study for the purposes of recruiting staff and students for the evaluation and will help to coordinate the assessments when the research team visits the school.

### Recruitment strategy

#### Implementation trial

Eligible school staff (see eligibility criteria table 1) who indicate willingness to participate in the trial at registration on the *TransformUs* website will be invited to click on a hyperlink connecting them to an online plain language description of the study, a consent form and a baseline survey.

**Table 1:** Inclusion and exclusion criteria for each trial

Target	Inclusion criteria	Exclusion criteria
Implementation trial		
School staff	Teachers, specialist support staff and other staff directly involved in teaching and learning activities, working in Australian government or independent schools located in Victoria (mainstream/special primary/secondary), Queensland or South Australia (special primary/secondary; mainstream secondary), who register for the *TransformUs* programme via the website.	Teachers, specialist support staff and other staff working at any Catholic school across Australia Teachers and other staff working at government or independent schools in New South Wales, Western Australia, Tasmania, Northern Territory and Australian Capital Territory will be able to register for the *TransformUs* programme but will not be invited to participate in the implementation trial. Other staff not directly involved in teaching and learning activities (eg, school administrative staff)
Effectiveness trial		
Schools	Up to six special government or independent special schools (primary, secondary or combined) located in Victoria catering for students with mild to moderate intellectual disability.	Mainstream schools Catholic schools Schools located in New South Wales, Western Australia, Tasmania, Northern Territory, Australian Capital Territory, Queensland, South Australia or outside Australia
School staff	School leaders: 1 nominated school leader per school (n=6) Teachers and teacher aides delivering the programme (approx. n=20)	School staff from schools not recruited for the trial
Students	Around 125 students with disabilities, both males and females, aged 6–18 years, attending one of the recruited schools. Focus groups (subsample): 18–30 students in total (max n=6 students per group)	Students younger than 6 years or older than 18 years.

#### Effectiveness trial

Principals or nominated school leaders will receive an invitation for their school to participate in the effectiveness trial. After receiving informed principal consent, schools will distribute recruitment information to parents/children. Parental written/electronic consent for their child to participate in the study will be sought prior to the study commencement. In addition to the informed consent received from parents/guardians, verbal assent from students will be obtained prior to any student assessment through any communication means (eg, verbally or via alternative and augmentative communication). Allocation to intervention/wait-list control will be done pragmatically, based on schools’ preference.

### Inclusion/exclusion criteria

The eligibility criteria for each study are outlined in table 1.

### Measures

An overview of the outcomes for the implementation-effectiveness trial is presented in table 2. More detailed information on each trial is available in the following sections.

**Table 2:** RE-AIM evaluation of the *TransformUs All Abilities* implementation and effectiveness trial

RE-AIM dimension	Measure/construct	Data source
Reach	Number and characteristics of registrants on the *TransformUs* website who indicate having children with disability in their classroom Estimated number of teachers and schools exposed to *TransformUs All Abilities* Relative representativeness of schools and students potentially exposed to *TransformUs All Abilities* (based on publicly available data on school characteristics and enrolments)	Web analytics (*TransformUs* website) Registration survey (*TransformUs* website) *MySchool* website (www.myschool.edu.au)
Effectiveness	Students Physical activity and sedentary behaviour (primary outcome) Sleep Physical literacy Cognitive functions (working memory) Students’ views on *TransformUs All Abilities*	Students Wrist-worn accelerometers (ActiGraph) Physical Literacy in Children Questionnaire (PL-C Quest—on iPad) Corsi Block-Tapping task (on iPad) Focus group discussions Teachers Teacher survey (pretrial and post-trial) Teacher interviews post-trial
Adoption	Reason for uptake of the programme Organisational readiness Organisational climate	Web analytics (*TransformUs* website) Registration survey (*TransformUs* website) Teacher survey (pretrial and post-trial) Teacher interviews post-trial
Implementation	Teaching practices and intention to implement Confidence, competence and barriers to implementing *TransformUs All Abilities* Teachers’ perceived barriers and facilitators to implementing *TransformUs All Abilities* Resources accessed (type, frequency) Strategies implemented by teachers (types, frequency)	Teacher survey (pretrial and post-trial) Teacher interviews post-trial Ecological Momentary Assessment (EMA) Web analytics (*TransformUs* website)
Maintenance	Teacher perception of potential to become part of routine educational practices Teacher views on factors affecting the long-term sustainability	Teacher survey (pretrial and post-trial) Teacher interviews post-trial

#### Implementation trial

**Figure 2:**
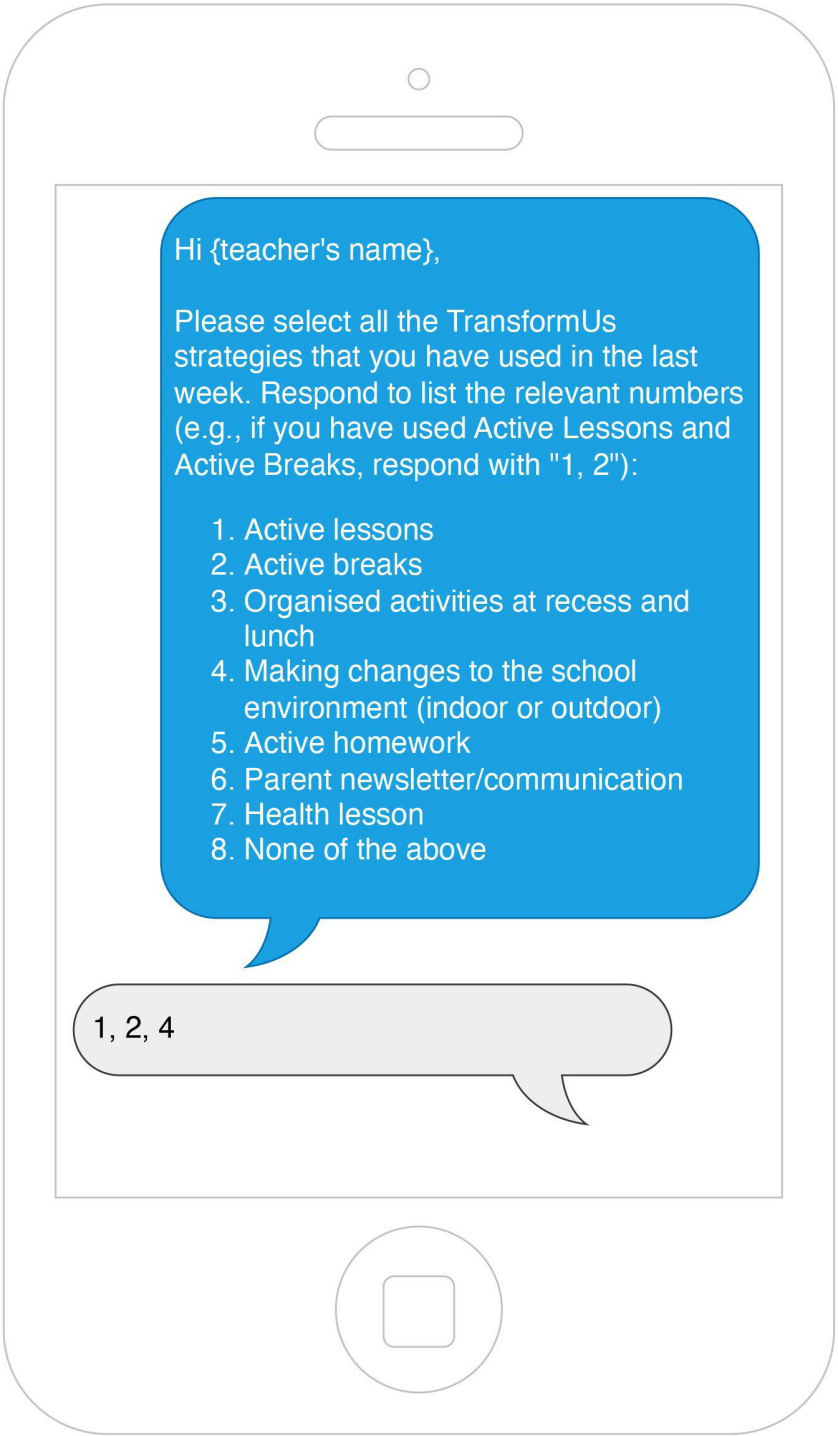
Ecological Momentary Assessment text messages used in the *TransformUs All Abilities* trial.

School staff’s demographic information, decision to register to the *TransformUs* website, their current teaching practices, organisational readiness, organisational climate, intention to implement the *TransformUs All Abilities* strategies will be collected at baseline using an online survey. Most constructs in the survey are measured using several items anchored to a 5-point Likert scale (strongly disagree—strongly agree). The online survey will be administered again 6 months after the registration to assess changes in school staff’s perceptions regarding *TransformUs All Abilities*. To assess frequency of intervention delivery, responses to up to three EMA text messages per school term will be sought for up to six school terms in total. Teachers will be asked to select from a list of seven strategies they used in the last week (figure 2).

To gather additional information on school staff’s experience with *TransformUs All Abilities*, a subsample of participants will be invited to participate in an interview at the end of 6 months. Interview questions will relate to participants’ experiences of delivering the programme, including any barriers or enablers. Throughout the trial, we will regularly download data that will indicate the number of All Abilities registrations to the *TransformUs* website. Website usage data, such as accessing All Abilities online resources, will be captured via Google Analytics.

### Effectiveness trial

#### Student outcomes

##### Baseline and post-trial

At baseline and at the end of the trial (approx. 20 weeks), students will complete assessments at the school in the presence of a school staff member to ensure that they feel comfortable in the environment and with people they are already familiar with.

Physical activity, sedentary behaviour (primary outcomes) and sleep (secondary outcome) will be measured using tri-axial GT9X ActiGraph accelerometers (ActiGraph, Pensacola, FL, SA). Students will be asked to wear them on their non-dominant wrist for eight consecutive days, including sleep and excluding water-based activities (eg, swimming or bathing) at both measurement points. The ActiGraph GT9X has demonstrated validity and reliability in children and adolescents.^59^ Data will be processed in R (https://transformus.com.au/) using the GGRI package, to determine time spent sedentary, in MVPA, and light, moderate and vigorous physical activity during school, recess, lunch and across weekdays and weekends. Sleep duration will also be analysed. A similar approach has been used in children with disability.^60^

Physical literacy (secondary outcome) will be assessed using the pictorial Physical literacy in Children Questionnaire (PL-C Quest).^61^ The Australian Physical Literacy Framework describes physical literacy as ‘lifelong holistic learning acquired and applied in movement and physical contexts’, which comprises 30 elements within four domains (physical, psychological, cognitive and social).^62^ The PL-C Quest is the only pictorial survey aligned with the Australian Physical Literacy Framework that uses images to depict each of the 30 elements. Each question contains two pictures with an orange bunny cartoon performing some activities. Children are asked to pick the picture where they think the cartoon character is being the most like them if they were in that situation. Once they pick that picture, they are asked how much the picture represents them (a bit or a lot). The scale has good internal consistency and evidence of construct validity in over 600 Australian children.^63^ In 2021, this scale was used in a dissertation with Canadian participants with intellectual and developmental disabilities; feedback from those participants and parents/guardians was very positive as to ease of use and enjoyment.^64^ An interactive version of the PL-C Quest will be administered using iPads. It takes approximately 4 min for children without disabilities to complete this assessment. It may require up to 8 min for children with disabilities to complete it, depending on their level of functioning. A researcher will help students to complete the survey, as required.

Cognitive functions will be measured using a forward Corsi Block-Tapping task (forward) powered by Inquisit Web (Milliseconds Software, LLC). Many versions of this task have been developed based on the original paradigm developed by Corsi.^65^ The version used in this study is an adapted version of the task developed by Kessels *et al.*^66^ The task presents a sequence of boxes lighting up in yellow in a predetermined (pseudo-random) order. After the last block is lit up, the participant is required to tap on the boxes that appeared in yellow, following the order in which they were lit. The task starts with a sequence of two blocks (level 2) and can continue up to nine blocks (level 9). Each level offers two chances to correctly reproduce the sequence of blocks. The test ends if a participant fails to reproduce two sequences of the same length. The assessment will be delivered via the Inquisit app on an iPad. Participants are provided with a practice session before starting the task. The approximate completion time is 2–4 min.

##### Post-trial only

To understand how students with disabilities perceived the intervention strategies, we will conduct a minimum of three 45 min focus group discussions with students (around n=6 students per group: 18–30 students in total). Trained researchers will guide the discussion which will be around students’ perceptions regarding the physical activity strategies implemented in the trial (enjoyment, physical/mental effort, favourite activities, perceived barriers, suggestions for improvements, etc). Similar discussions will also be held with students in the wait-list control schools to learn more about students’ current experiences at schools in relation to movement-based activities, their preferences and potential interest in the types of activities included in the *TransformUs All Abilities* programme. All discussions will be audio recorded and later transcribed verbatim for analysis. Drawings or notes produced during the focus groups will be collected or photographed.

### School staff outcomes

School staff will complete the same survey described in the implementation trial section at baseline and 6 months later. Teacher responses to EMA text messages sent up to three times per term for the duration of the trial to the three intervention schools will be collected as a measure of fidelity. Teachers from the three intervention schools will be invited to participate in an interview at post- aimed at learning more about their experience with the programme.

### Sample size and power calculation

#### Implementation trial

Based on reach, we aim for at least 52 special schools (ie, 25% of the 208 eligible special schools) and 98 mainstream schools (ie, 5% of the eligible 1965 mainstream primary/secondary/combined schools) to uptake the *TransformUs All Abilities* programme.

#### Effectiveness trial

For our primary outcome—ActiGraph-measured MVPA (min/day)—we are aiming to recruit a total of 134 students (intervention n=67; control n=67). Allowing for a 30% drop-out and incomplete data (accelerometer not worn for enough hours per day or days over the week), this would still give us a sufficient power to detect statistically significant differences in the primary outcome.

For data to be analysed with linear mixed models (additional details available in section ‘Data Analysis’), the fixed effects were estimated with an a priori power calculation using G*Power software. Assuming a moderate effect size (f² = 0.15), an α error probability=0.05, and power (1 – β error probability) = 0.80, and the group variable as predictor, fixed effects were estimated to require 55 observations. To account for the random effects of clustering (students in the same classroom), a design effect of 1.82 was estimated (based on a coefficient of variation of 0.15 and an average number of five students per classroom and a conservative Intraclass Correlation Coefficient value of 0.20), suggesting that 100 observations would be required (ie, 55×1.82 = 100). Accounting for 25% attrition, the final total sample size needed for the effectiveness trial is 134 participants (ie, 100 ÷ (1–0.25) = 134), from 27 classrooms (134 ÷ five students per class=27).

### Timeline

The implementation trial will start in mid-2024 and participants will be able to enrol to the study up until the end of June 2025. Any school staff who registers after the end of June 2025 will not be invited to take part in surveys or interviews, as subsequent follow-up would exceed the implementation trial end date. The parallel effectiveness trial will be conducted for approximately 6 months (ie, 20 weeks); eligible schools will be able to register in the trial between mid-2024 and mid-2025 for a staggered and matched (intervention—wait-list control) start and a maximum duration of the trial not exceeding the end of 2025.

### Data analysis

Qualitative information collected from the interviews and focus groups will be analysed using thematic analysis^67^ in NVivo V.14 (released in 2023, Lumivero). Using a hybrid inductive/deductive approach,^68 69^ we will code data inductively and categorise codes in themes according to common implementation outcomes (ie, acceptability, adoption, feasibility, fidelity, penetration, sustainability and satisfaction).^57^ Where coded quotes do not apply to any of the preconcerted themes, new themes will be created.

Quantitative data will be analysed using Stata V.18 (Stata Corp. LLP). Preliminary analysis will be conducted to describe the sample (demographic characteristics and distribution of the scores on the outcomes of interest). Teacher-level outcomes from survey and EMA text message responses—including current teaching practices, organisational readiness/climate, teacher’s competence/confidence, and perceived barriers and facilitators in implementation, as well as frequency and types of *TransformUs All Abilities* strategies implemented by teachers (ie, EMA responses)—will be summarised descriptively. Changes over time in those outcomes’ scores will be analysed using linear mixed models. For teachers in the implementation trial, we will use time as a predictor variable (baseline/follow-up for survey scores; multiple time points for EMA). For teachers participating in the effectiveness trial, the predictor of interest will be the interaction between study group (intervention/control) and time. All models will be adjusted for common confounders (ie, teacher’s age, sex, years of teaching experience) and controlled for the random effect of classroom as a clustering variable.

Student-level primary and secondary effectiveness outcomes will be analysed using separate linear mixed models to assess the fixed effects of the intervention (ie, study group [intervention/control] × time [baseline/follow-up]) on each continuous outcome (ie, physical activity, sedentary time, sleep, physical literacy and cognitive functions), adjusting for common confounders (ie, age, sex and student disability/functioning) and controlling for the random effects of clustering (ie, students in classrooms). The effects of an interaction term between study group, time and student disability/functioning will also be explored, to see whether different levels of support moderate the intervention effects.

A person-centred analysis will also be explored as secondary analysis. Person-centred analyses involve creating groups of participants with similar characteristics or similar scores on the independent variables using a variety of techniques (eg, Cluster analysis; latent class analysis) to reduce the total variability.^70^ compared with variable-centred approach, this method has been suggested as a possible way of exploring how participants grouped by similar characteristics respond differently to the intervention in relation to the main outcomes of interest.^70^

## Ethics and dissemination

### Ethics approvals and trial registrations

This research has received ethical clearance from the Deakin University Human Research Ethics Committee on 16 December 2021 (2021-368). Permission to conduct research in schools has also been provided by the Education Departments in Victoria (31 March 2023, ref. 2023-004726), Queensland (11 July 2022, ref. 550/27/2592) and South Australia (1 September 2022, ref. 2022-0020). Prospective trial registrations were completed for the implementation trial (ACTRN12622001082796; Universal Trial Number: U1111-1281-1103) and effectiveness trial (ACTRN12622001050741; Universal Trial Number: U1111-1280-8828) through the Australian New Zealand Clinical Trial Registry (ANZCTR) on 4 August and 28 July 2022, respectively. Voluntary opt-in informed consent is required to take part in this study. Eligible school staff can consent to take part in the implementation trial via the *TransformUs* website. For the effectiveness trial, school leaders must provide informed consent for their school to participate; schools will then seek written or electronic parental/carer consent for their child’s participation and students will provide verbal assent before any assessments. Examples of the participant consent forms are provided in online supplemental file 2. This research will be performed in accordance with the standards of ethics outlined in the Declaration of Helsinki.

### Data management

Audio recorded interviews/discussions will be transcribed verbatim and identifiable information will be removed prior to the analysis. Transcripts will be sent to participants to allow them to review or edit their responses (member checking). Physical activity, sedentary behaviour and sleep data will be downloaded from the accelerometers using ActiLife software and kept in shared folders only accessible by the research team. Data will then be processed in RStudio using the GGIR package.^71^ Published equations will be used to identify time spent in sedentary, light physical activity and MVPA during waking hours.^60 72 73^ Sleep will also be extracted based on previously published methods.^74^

### Dissemination

Plans for dissemination include sharing results with participating schools, parent groups and disability organisations through tailored summaries, presentations and accessible formats (eg, plain language reports, presentations at relevant non-scientific events and webinars). Participants and relevant representatives of the public will be consulted on preferred formats and timing for dissemination. Additionally, results will be disseminated with the wider scientific community via academic publications and conference presentations.

## Supplementary material

10.1136/bmjopen-2025-105311 online supplemental file 1

10.1136/bmjopen-2025-105311 online supplemental file 2
