# Supplementary material for: School-based strategies to increase physical activity and reduce sedentary behaviour in students with disability: protocol of the TransformUs All Abilities hybrid type II implementation-effectiveness trial
Source: BMJ Open. 2025 Nov 9;15(11):e105311. doi: 10.1136/bmjopen-2025-105311 (PMC12598974; doi:10.1136/bmjopen-2025-105311)
Supplement: online supplemental file 1 [file bmjopen-15-11-s001.pdf]

Supplementary File 1. Examples of adapted TransformUs resources

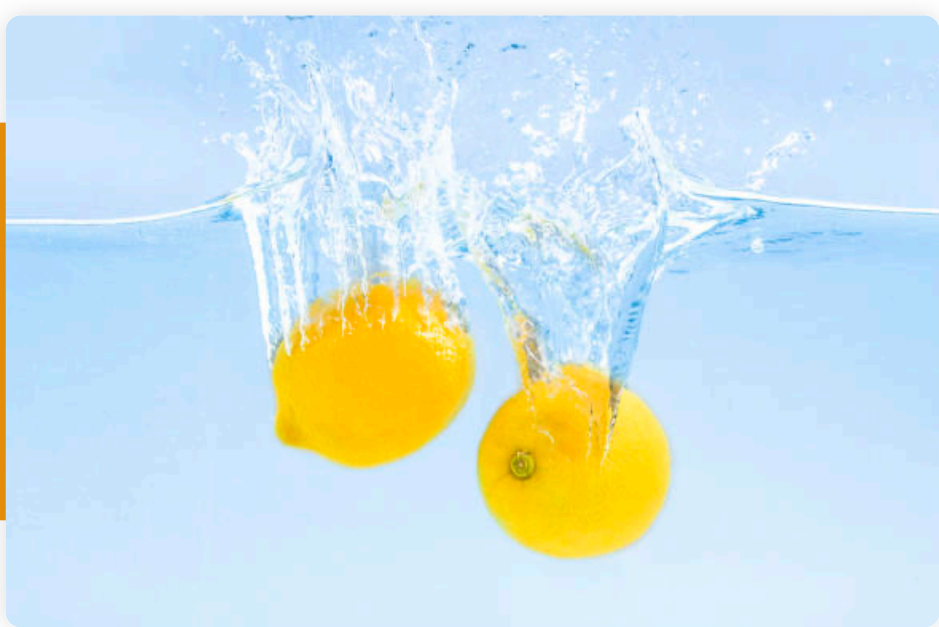

Source: Envato Elements

Back to resources →

Active Lesson

Sink or Float?

Questioning and predicting

Curriculum Codes (VIC): VCSSU044, VCSIS050, VCSIS051, VCSIS052, VCSIS053
Curriculum Codes (AU): ACSSU003, ACSHE014, ACSIS024, ACSIS037, ACSIS011, ACSIS025, ACSIS038, ACSIS026, ACSIS039, ACSIS027, ACSIS040

Year Level:
Foundation, Year 1 - 2

Setting:
Classroom, Outside

Subject:
Science

Strands:
Science understanding
Science inquiry skills

Sub-Strands:
Physical sciences
Questioning and predicting
Planning and conducting
Recording and processing
Communicating

Experiment:
Yes

All Abilities:
Yes

Learning Intention

We are learning why objects sink or float.

Success Criteria

- I know objects are made of molecules.
- I know a dense object has molecules close together.
- I know a light object has molecules spread out.
- I can describe density and buoyancy.
- I can describe my observations throughout the experiment.

Description

1. Discuss density and buoyancy with the class: Before explaining why items sink or float, ask students why they think an object sinks or floats. They may guess it is because of size, weight or construction. Then, explain that items sink or float based on their density. Density is determined by how close or far apart molecules are within an item. Molecules are tiny and only visible by a microscope. See further explanation below.

2. Explain that in this activity, students will need to make predictions and observations about buoyancy and density. Through their observations, they will learn that buoyant objects float, and dense objects sink, they should record their predictions and observations before the experiment.

3. Fill a tub or bucket with lukewarm water. Show students the activity items and ask them to predict and record which objects they think will sink and which will float. Alternatively, this can be done by a show of hands in favour of an item sinking or floating. If you are learning about tally marks in mathematics, construct a tally beside each item to indicate student's hypothesis.

4. Select students to drop the objects, one by one, into the water. Students should observe and record what happens to each object.

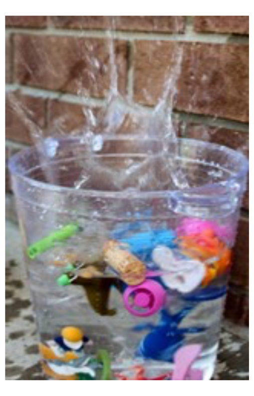

Note: If you are doing this experiment indoors, place a plastic tablecloth or some beach towels under the container to soak up any spills.

Equipment

- Water
- Waterproof toys and household items (see suggested items below)
- A container to do the experiment in i.e., plastic tub, pot, bucket etc

Science Explained

Why do things float?

Everything is made of molecules. Molecules are very, very tiny. You can only see them with a special microscope. In some objects, like a rock, molecules are squished tightly together. In others, like wood, the molecules are more spread out. How closely molecules are crowded together in a space is called density. Density is a big part of why some things float and others do not. Objects like coins, rocks, and marbles are denser than water. They will sink. Objects like apples, wood, and sponges are less dense than water. They will float. Many hollow things like empty bottles, balls, and balloons will also float. That is because air is less dense than water. This is one reason huge ships can float, even though they are very heavy. Inside a ship, there is a lot of hollow space filled only with air. But that is not all: shape matters, too!

Examples

Suggested Items

Items that Sink: metal utensils, coins, stones, toy car/truck, keys, marbles, glass, gemstones.

Items that Float: Duplo/Lego, sticks or popsicle sticks, corks, bath toys, foam shapes, crayons, rubber balls, sponge, wood, empty bottles.

Extensions

Once all the items are in the water, extend the activity by giving the students drinking straws to blow the floating items around. This is a fun way to teach students about kinetic energy as the air they blow through the straw propels the floating items forward.

All Abilities

Sensorial

Teaching style

- Explain the task in a clear and simple manner. While explaining the concepts of buoyancy (i.e., floating property) and density, you could allow students with a vision impairment to handle each object so they can get familiar with their shape and weight.
- To ensure students with a hearing impairment have access to simple and clear information on the concepts of buoyancy and density you could use a video explanation with closed captions played for the whole class, and/or an infographic-style resource.
- Consider learning how to sign key words such as sink and float in AUSLAN (click the hyperlinks to find out how). Search how to sign many other words by clicking here.

Rules and conditions of the task

- Allow for different ways to present information with tables and drawings. You can improve engagement by working with students to identify methods that work for them. For instance, allow students with a vision impairment to use their assistive device with speech-to-text capability.
- Encourage students with vision impairment to work independently by allowing them to manipulate the objects prior to asking questions to the whole class.

Equipment

- Ensure students have access to any Augmentative and Alternative Communication (AAC) devices that they may need to communicate their thoughts and/or recording classwork during the activity.
- You could place a tactile object in front of the bucket so students with a vision impairment know where to stop independently. Run a few trials at the start to make sure students have some points of reference and are familiar with the distance from the water.

Environment

- Ensure the floor stays dry, as this may present as a slipping hazard.
- Arrange the table/workstation so that students have a clear view of the container and the objects being dropped in and the teacher.
- Ensure noise is maintained to a minimum to ensure students with vision impairments can follow what is being discussed.

Physical

Cognitive

Social-Emotional

Source

https://happyhooligans.ca/sink-or-float-experiment-preschoolers/

Image Source
https://happyhooligans.ca/sink-or-float-experiment-preschoolers/

Related resources

Search all resources →

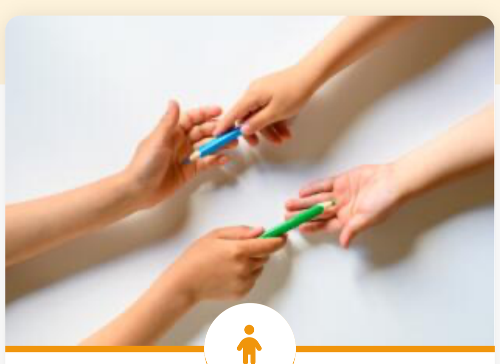

ACTIVE LESSON
Any Topic
Solve and Switch (English)
Provide each student with a task card with a literacy problem.
ENGLISH | ANY YEARS
ADAPTED

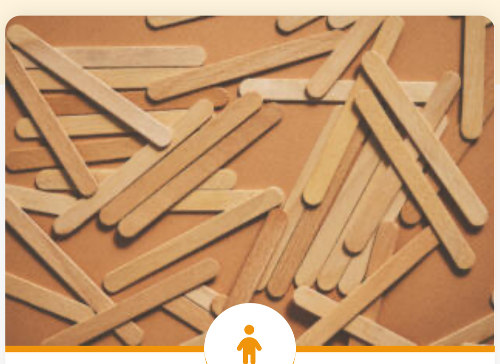

ACTIVE LESSON
Number and place value
Whole Number Pop Sticks
In teams of four or five, students stand five to ten metres away from a set of 10 bundles of paddle pop sticks.
MATHEMATICS | YEAR 1 - 2
ADAPTED

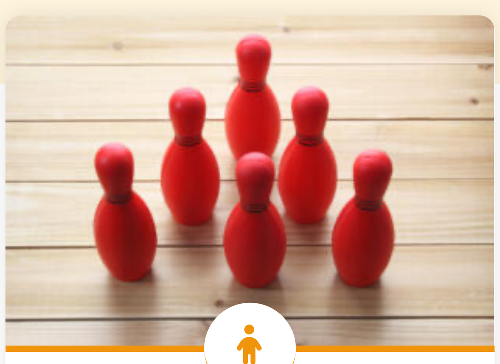

ACTIVE LESSON
Number and place value
Math Bowling
Arrange the class into small groups. Ask each group to set up their skittles either in a traditional triangle or in a group.
MATHEMATICS | FOUNDATION, YEAR 1 - 3
ADAPTED

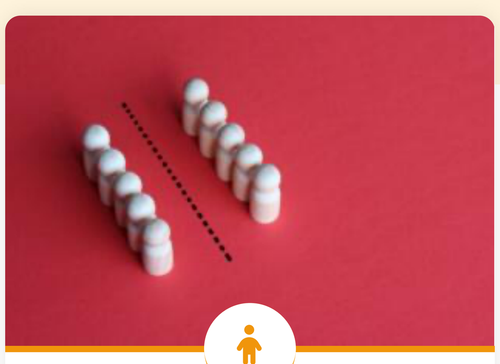

ACTIVE LESSON
Number and place value
Dividing People
Provide the class with a division equation, e.g., 25 ÷ 5 =
MATHEMATICS | YEAR 2 - 5
ADAPTED

© Copyright Deakin University 2025. Deakin University CRICOS Provider Code: 00113B

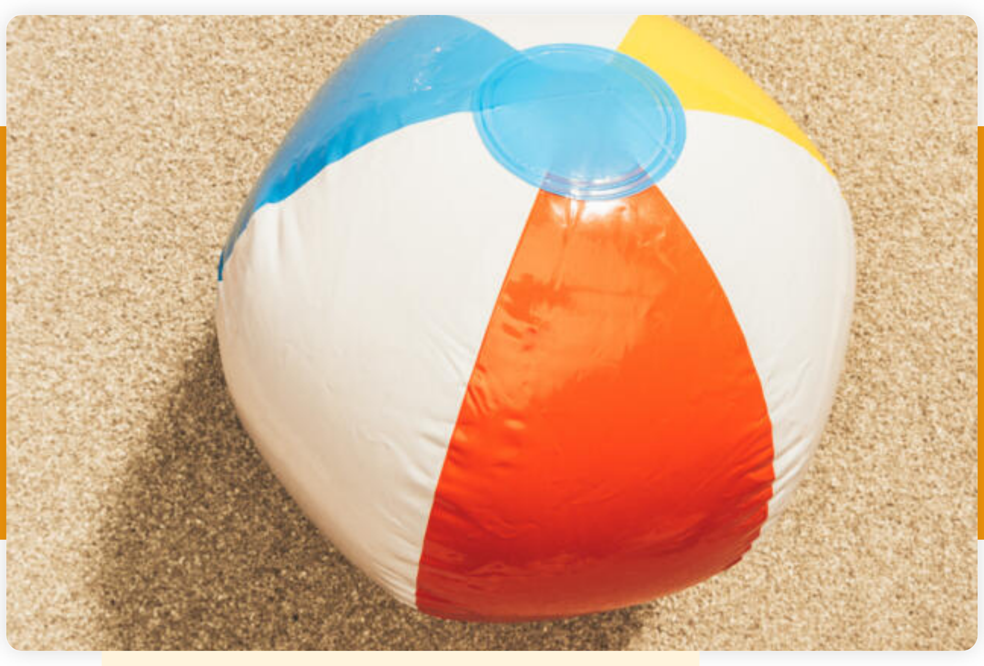

Source: Envato Elements

Back to resources →

Active Lesson ?

Prefix and Suffix Catch

Phonics and word knowledge

Curriculum Codes (VIC): VCELA250, VCELA312, VCELA354
Curriculum Codes (AU): ACELA1827, ACELA1779, ACELA1828, ACELA1514, ACELA1513

Year Level:
Year 3 - 6

Setting:
Classroom, Outside

Subject:
English

Language Mode:
Reading and viewing
Writing

Strands:
Language

Sub-Strands:
Phonics and word knowledge

All Abilities:
Yes

Learning Intention

We are learning about prefixes and suffixes.

Success Criteria

- I know that a prefix is placed at the start of a word.
- I know that a suffix is placed at the end of a word.
- I know the meaning of a new word.

Description

1. Write various common prefixes and suffixes on a beach ball.

Prefix examples; anti-, dis-, in-, mid-, non-, semi-, trans-, un-, sub-, mis-

Suffix examples; -able, -ful, -less, -ness, -ment, -ing, -est, -tion, -ious

2. Ask students to stand in a circle.

3. Students throw the ball to their classmates, wherever their hand lands, they have to say a word using that prefix/suffix.

4. The student then spells the word and/or says the word in a sentence.

5. Students get a point for every correct word, and half a point for spelling the word correctly and/or using it in a sentence.

6. If a student cannot think of a word, they run a lap of the circle. While running, they tap another student on the shoulder nominating them to attempt a word using the prefix/suffix.

Equipment

- Beach ball
- Permanent marker
- A clear, open space

Extensions

Prefixes and suffixes meanings

Students can also attempt the meaning of the prefix/suffix, e.g.,:

anti- = against

dis- = not, opposite of

mid- = middle

non- = not

semi- = half

trans- = across

un- = not

mis- = wrongly

-able = can be done

-ful = full of

-less = without

-est = comparative

-tion = act, process

-ious = possessing the qualities of

Homework

Students go for a walk around their house, backyard or neighbourhood. They identify five objects. Students write a sentence about each object, each sentence must include at least one word with a prefix or suffix. Lastly, students attempt the meaning of the prefixes/suffixes used.

All Abilities

Sensorial

Physical

Teaching style

- Provide alternative actions for Step 2 and 6 to meet the varying abilities within your class. For example, if students are unable to stand for Step 2 they could sit and instead of walking or running a lap of the circle for Step 6 they could perform arm circles or another more accessible movement instead.

Rules and conditions of the task

- Demonstrate the alternative ways to complete the task. For example, show how the task will look if it is going to be completed with some students remaining in one place if they have limited mobility. Rules may also be modified to allow other students to move around the circle for them instead.
- Allow alternative actions and movement patterns (walk) if running is not accessible.

Equipment

- Provide a bell ball if available and relevant.
- Have whiteboard available to record student responses.
- Provide chair if student requires to complete activity seated.

Environment

- Consider where you conduct the activity to support students with limited mobility.
- Ensure activity space is accessible for students with limited mobility. Ensure a flat, safe workspace is provided with no trip hazards.
- Modify the distance students are required to run around the circle if this is too challenging.
- Students could be passed the ball from a closer distance if it is too challenging from a greater distance.

Cognitive

Social-Emotional

Source

Modified from:
https://minds-in-bloom.com/15-ways-to-teach-prefixes-and-suffixes/

Related resources

Search all resources →

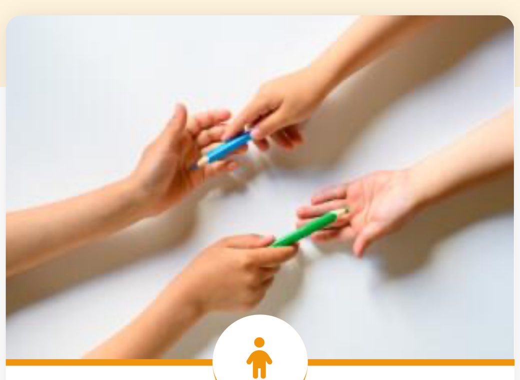

ACTIVE LESSON
Any Topic
Solve and Switch (English)
Provide each student with a task card with a literacy problem.
ENGLISH | ANY YEARS | ADAPTED

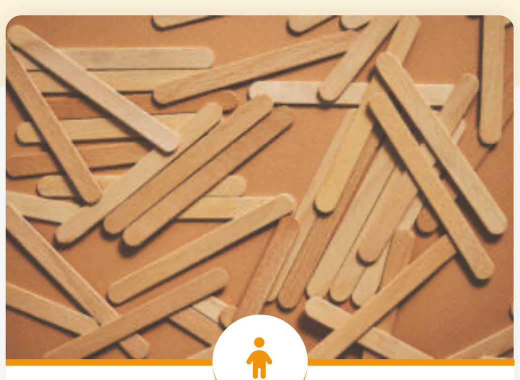

ACTIVE LESSON
Number and place value
Whole Number Pop Sticks
In teams of four or five, students stand five to ten metres away from a set of 10 bundles of paddle pop sticks.
MATHEMATICS | YEAR 1 - 2 | ADAPTED

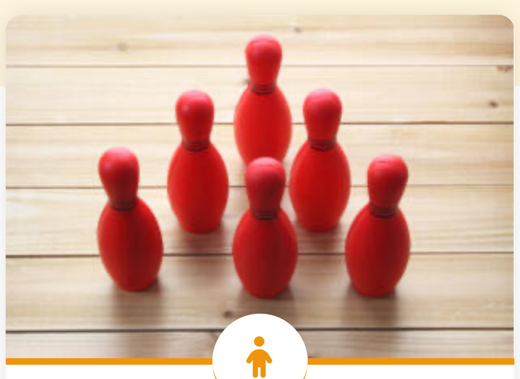

ACTIVE LESSON
Number and place value
Math Bowling
Arrange the class into small groups. Ask each group to set up their skittles either in a traditional triangle or in a group.
MATHEMATICS | FOUNDATION, YEAR 1 - 3 | ADAPTED

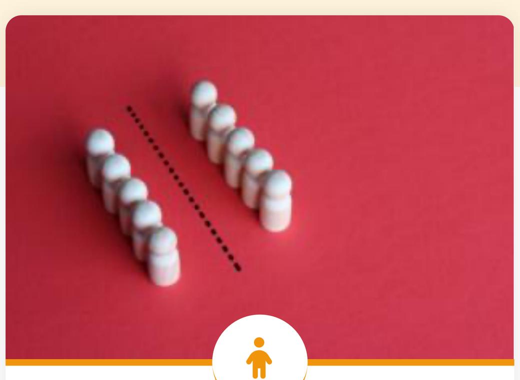

ACTIVE LESSON
Number and place value
Dividing People
Provide the class with a division equation, e.g., 25 ÷ 5 =
MATHEMATICS | YEAR 2 - 5 | ADAPTED

© Copyright Deakin University 2025. Deakin University CRICOS Provider Code: 00113B

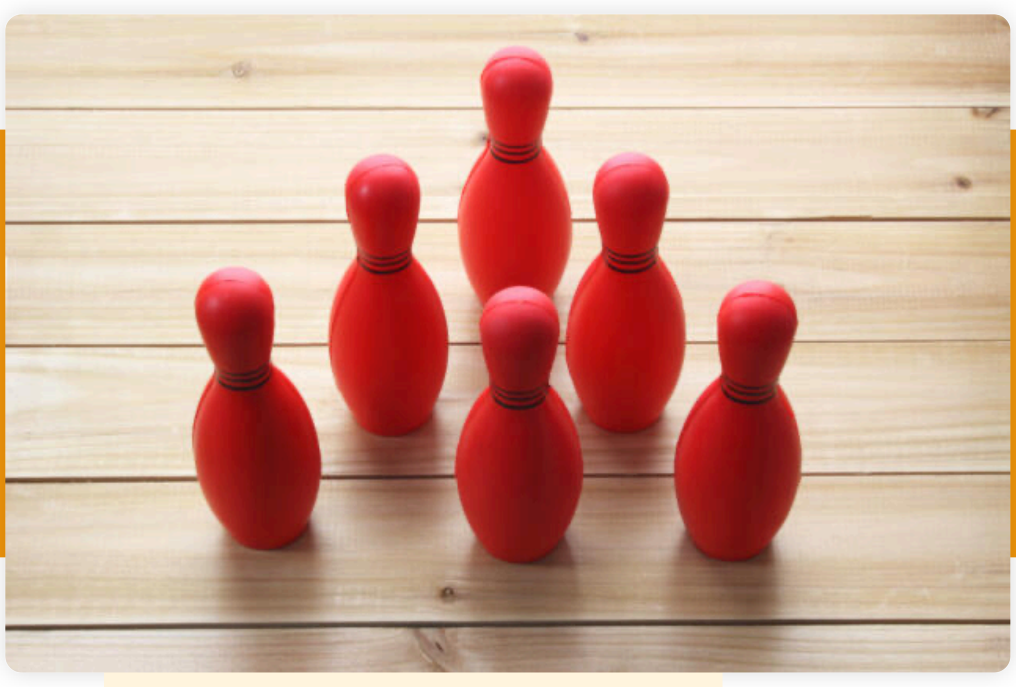

Back to resources →

Active Lesson

Math Bowling

Number and place value

Curriculum Codes (VIC): VCMNA086, VCMNA088, VCMNA089, VCMNA106, VCMNA107, VCMNA132, VCMNA133
Curriculum Codes (AU): ACMNA012, ACMNA014, ACMNA015, ACMNA029, ACMNA030, ACMNA054, ACMNA055

Source: Envato Elements

Year Level:
Foundation, Year 1 - 3

Setting:
Outside, Classroom

Subject:
Mathematics

Strands:
Number and algebra

Sub-Strands:
Number and place value

All Abilities:
Yes

Learning Intention

We are learning to subitise numbers to 10.

Success Criteria

- I can subitise numbers up to 10.
- I can check my answers using subtraction.

Description

1. Arrange the class into small groups. Ask each group to set up their skittles either in a traditional triangle or in a group.

2. Set the bowling distance at least 10 steps away from the skittles. Use masking tape to create a bowling line.

3. Students bowl the ball towards the skittles, aiming to knock as many over as they can.

4. Using the skittles knocked down and the skittles still standing, review and practice subitising, e.g., there are 7 skittles knocked down and 3 skittles left standing, therefore 10 – 7 = 3.

5. Repeat the process for students to practice subitising.

Equipment

- Sets of numbered skittles or empty plastic drink bottles
- Small balls e.g., tennis ball
- Masking tape
- Notebooks and pens

Variations

1. Students repeat the activity, this time they record the answers for each bowl on a sheet of paper and then add the answers cumulatively. The aim is to get to 50. Students record how many attempts it takes to get to 50.
2. Students repeat the activity; this time they start at a score of 50 and subtract their answers after each bowl from the previous score. Students subtract down to zero and record how many attempts it takes to get to 0.
3. Students repeat the activity; this time using the numbers on the skittles thus larger numbers will be generated. Select a higher target e.g., 300.
4. Students repeat the activity; after each ball, ask the students to order the digits they knocked over to create the largest number possible. The winner is the individual who achieves the largest total.

Homework

Students can complete this activity at home using 10 items to resemble ten pin bowling pins (e.g., empty plastic milk, water or juice containers, wooden blocks, toy statues), tennis ball or rolled up socks to bowl with and five meters of flat clear space – a passage way is ideal!

All Abilities

Sensorial

Physical

Cognitive

Teaching style

- Provide clear and simple instructions for the task. You can even try using a diagram on the whiteboard to explain how the activity will unfold and provide examples.
- Break down the activity in different simple parts [e.g., playing bowling; writing numbers (1-10) on the whiteboard counting the falling skittles following the order presented on the whiteboard]. Ensure that students can become familiar with each part before combining everything.
- Have several practice runs, talking students through the task, and use some simple questions to verify that students understand the task.
- If a student uses PECS, they can communicate key tasks in the activity using cards signifying 'ball', 'roll', and cards that relate to the equations being taught.

Rules and conditions of the task

- Consider using additions instead of subtractions for student who may find it very challenging to complete these operations.
- If a student has a preferred way to bowl the ball, let them do so if it is safe for the class. For example, they might want to kneel and push the ball instead of stand and roll.
- Students can line up to take turns bowling. This may help make it clear when it is a student's turn and reduce anxiety.

Equipment

- Some students may not be able to bowl with a bigger/smaller ball. Think about providing balls of different sizes so that students can pick the one they feel most comfortable with. If skittles get knocked over too easily, you could experiment adding weight to them with water or rice, which may be an exciting experiment on its own for students.
- You can add the number '1' to each bottle. This may help facilitate independence when solving the equations. Use bright masking tape to mark important locations of the task, for example where to stand, this may help reduce cognitive load.

Environment

- Try to foster a positive and inclusive environment. For example, as this is a group activity, some students may become embarrassed or upset if they get an equation wrong. When explaining how to correctly solve an equation, focus on explaining to the class, and not focusing on the student.
- Ensure the area has adequate lighting for students to bowl.
- Whether you conduct this activity in a classroom or outdoors, try to ensure the location is familiar to students.

Social-Emotional

Source

https://www.teachstarter.com/au/teaching-resource/ten-add-bowling-active-learning/

Related resources

Search all resources →

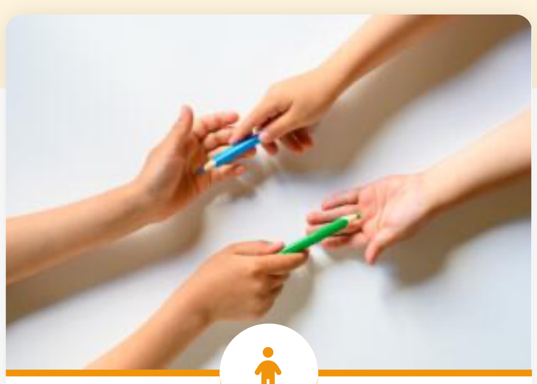

ACTIVE LESSON

Any Topic
Solve and Switch (English)

Provide each student with a task card with a literacy problem.

ENGLISH | ANY YEARS | ADAPTED

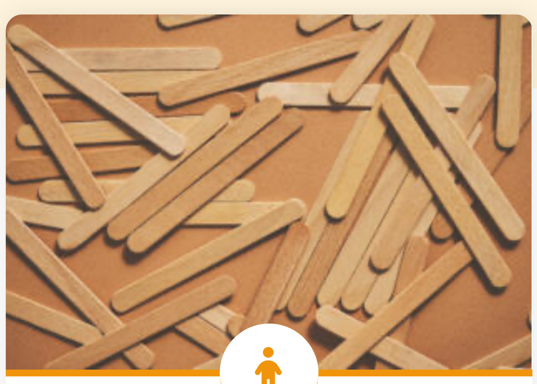

ACTIVE LESSON

Number and place value
Whole Number Pop Sticks

In teams of four or five, students stand five to ten metres away from a set of 10 bundles of paddle pop sticks.

MATHEMATICS | YEAR 1 - 2 | ADAPTED

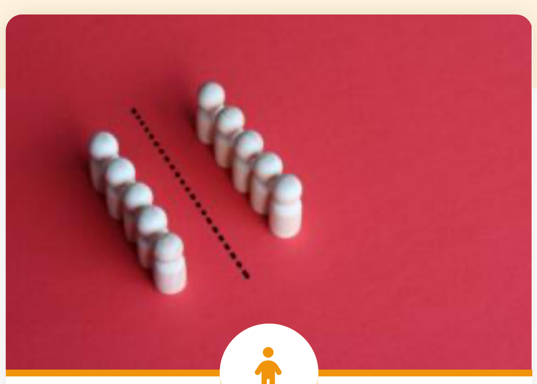

ACTIVE LESSON

Number and place value
Dividing People

Provide the class with a division equation, e.g., 25 ÷ 5 =

MATHEMATICS | YEAR 2 - 5 | ADAPTED

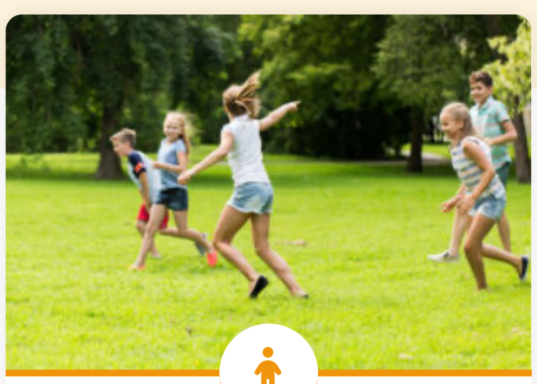

ACTIVE LESSON

Any Topic
Math Tails

Create and laminate some Math tails on strips of paper (see examples).

MATHEMATICS | YEAR 1 - 6 | ADAPTED

© Copyright Deakin University 2025. Deakin University CRICOS Provider Code: 00113B

Privacy | Terms of use

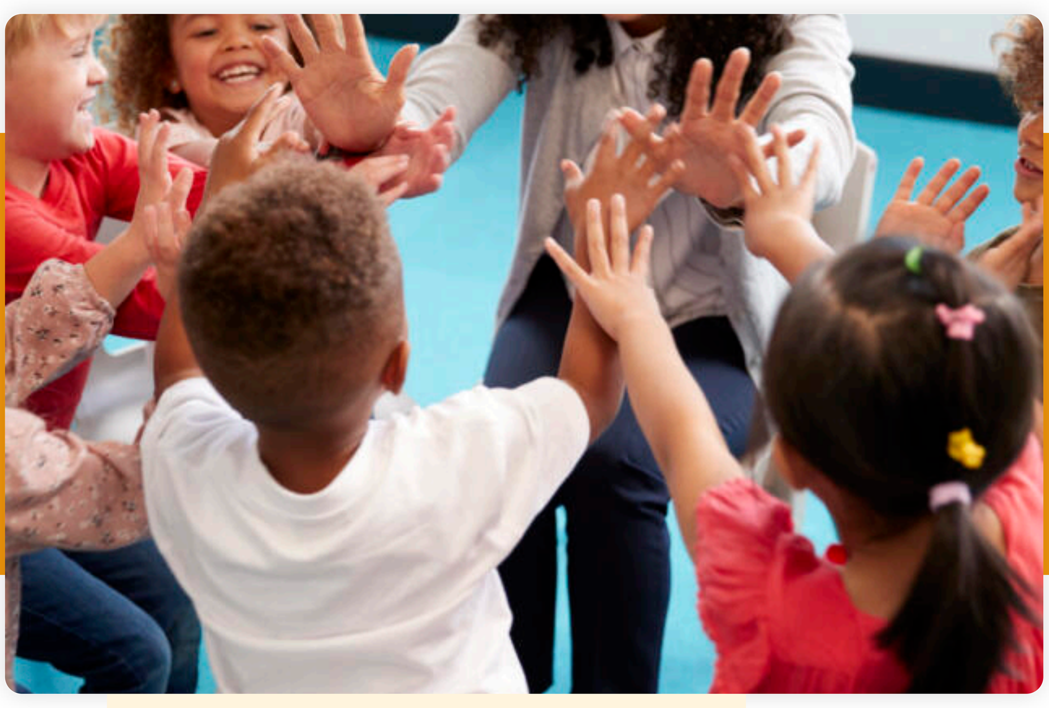

Back to resources →

Active Break

Compliment Tag

Energise, Transition

Source: Envato Elements

Year Level:
Foundation, Year 1 - 4

Activity Intensity:
Moderate

Curriculum Linked:
No

All Abilities:
Yes

Aim

To get students active while encouraging positive interactions and communication among peers.

Energise: The purpose here is to break up periods of prolonged sitting through the provision of a physical activity. The Energise Active Break enables students to become re-energised, refocused and re-engaged in their learning.

Transition: The purpose of this Active Break is to allow intentional, task orientated movement as students transition between one learning task or phase of the lesson, to the next.

Description

When the music starts, students move around the room by skipping, side stepping, walking, jumping, hopping, marching, dancing or via a movement of their choice. Once the music stops, they freeze and face a peer, raising both hands. They 'high ten' with both hands, or gently touch elbows, and offer a compliment to their partner. Then, as the music starts again they begin to move with a newly selected form of movement, once the music stops they turn to a new person and repeat the process of high ten and compliment. Repeat as many times as you feel appropriate. Teachers may like to use this Active Break during a transition period in the lesson, when students are moving from one physical location e.g., from the floor/mat, to another physical location in the room e.g., their desks.

PRACTICAL USE

This Active Break is intended to promote energy, enthusiasm and engagement after lengthy periods of sedentary learning. This Active Break would be used during extended seated learning tasks to break prolonged sitting. This Active Break can also be used as a 'Manage' Active Break, if used in a proactive manner (prior to noticing declines in energy and focus) and by reducing the intensity of the movement (if necessary).

This Active Break can also be used during the transitions within the lesson. This Transition Active Break is goal orientated and purposeful as it allows students to move from where they are in the learning/lesson, to where they need to be in the next phase of the learning/lesson. In this case, the Active Break aims to transition students from one physical location to another (e.g., the mat to desks)

Equipment

- Music

Variations

Silent Handshake

Students silently choose a number between one and five on their own. The goal is for students to find others who picked the same number. They move around the room, shaking hands with other students – no talking allowed. They shake the same number of times as the number they selected. When they find someone with the same number, they link arms and continue to walk slowly around the room. New students can join their group if their numbers match that of the group.

Once all groups have formed, have students freeze and ask each group to reveal their number.

For an added challenge, you can ask one group to state another group's number or have the groups silently arrange themselves in order from one to five across the room.

All Abilities

- Sensorial
- Physical
- Cognitive
- Social-Emotional

Teaching style

- Let students know about this active break in advance. Outline what the activity entails and focus on the positive nature of compliments, as the idea of receiving comments from fellow students may be uncomfortable for some.
- You could privately ask each student if there is anything they do not want others to talk about. You could then turn those into generalised rules that apply to everyone. For example, "We do not compliment on students' hair colour/style".

Rules and conditions of the task

- If students are not comfortable with physical contact, suggest alternative methods, such as a wink, or nodding your head to left or right.
- Before starting the task, ensure you clearly outline what a compliment is, as you will want to avoid students who may misinterpret the activity and say something upsetting.
- Provide students with additional time to find their partner when the music stops.

Equipment

- Only music is needed for this activity. Some students may be very sensitive to sound but may still want to engage in this activity. Try to ensure that these students have access to noise cancelling headphones.
- You could ask students if there are any songs or type of music they would like to listen to during the activity. Playing songs that are familiar to the students may help reduce anxiety.

Environment

- You may need to adjust the volume of the music to ensure students can hear it but is not overbearing for those who are sensitive to sound/noise.
- Ensure students are spread out. This may help to reduce social and spatial overwhelm.
- Sometimes things do not always go the way we want, and students may become distressed. In this case, ensure the students can easily access a quiet space in the school premises to calm down.

Related resources

Search all resources →

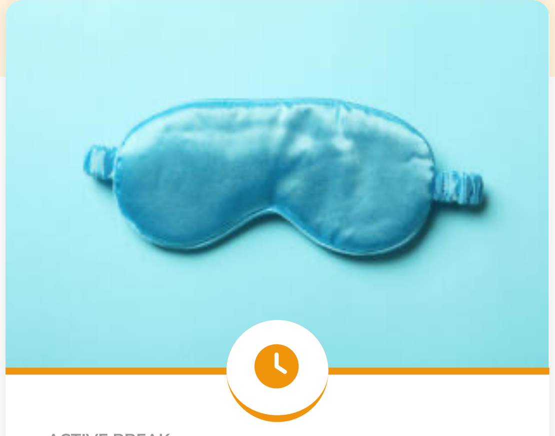

ACTIVE BREAK
Energise, Manage
Where Did the Class Go?
To break from long periods of seated learning by playing a game that involves sneaking, silence, listening and guessing.
FOUNDATION, YEAR 1 - 4

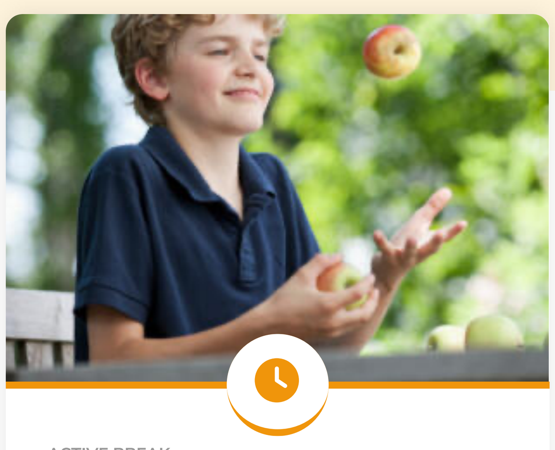

ACTIVE BREAK
Structure, Energise
Group Ball Juggling
To practise Math learning concepts such as patterns, numbers and skip counting, in a physically active way.
MATHEMATICS — FOUNDATION, YEAR 1 - 6

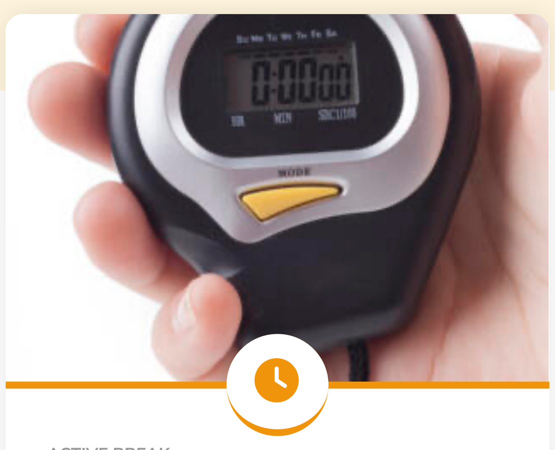

ACTIVE BREAK
Learn, Structure
Count Your Steps
To generate numerical data (through the use of physical activity), that can be collected, represented and manipulated.
MATHEMATICS — YEAR 3 - 6

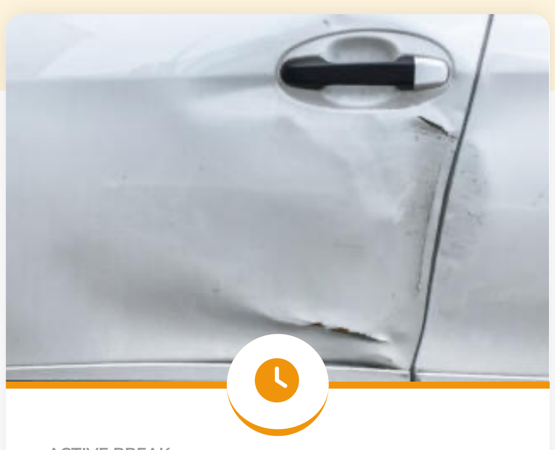

ACTIVE BREAK
Transition, Energise
Don't Dent the Car
To transition from independent/teacher-led learning to collaborative learning groups using a physically active game and to break up...
FOUNDATION, YEAR 1 - 2

© Copyright Deakin University 2025. Deakin University CRICOS Provider Code: 00113B
