# Supplementary material for: School-based strategies to increase physical activity and reduce sedentary behaviour in students with disability: protocol of the TransformUs All Abilities hybrid type II implementation-effectiveness trial
Source: BMJ Open. 2025 Nov 9;15(11):e105311. doi: 10.1136/bmjopen-2025-105311 (PMC12598974; doi:10.1136/bmjopen-2025-105311)
Supplement: online supplemental file 2 [file bmjopen-15-11-s002.pdf]

Supplementary File 2. Plain Language Statements and consent forms

TransformUs All Abilities

DEAKIN UNIVERSITY

Plain Language Statement for Implementation Trial

Overview of the project

TransformUs is a learning through movement evidence-based initiative that has been running in Victorian primary schools for over 10 years. Teachers have told us that their students have shown better focus in class and classroom management has improved. We have also observed increases in movement and decreases in sedentary time, as well as health and behavioural benefits in the primary school setting. TransformUs has now been adapted to cater for the additional needs of students with disability and this project aims to test if the program has similar benefits in these students. The aim of this project is to understand whether the TransformUs program adapted to the specific needs of children with disabilities is feasible, practical, and sustainable in the school environment.

This project is being led by Professor Jo Salmon at Deakin University and is funded by the National Health and Medical Research Council.

What is TransformUs?

TransformUs is designed to re-frame the way students learn by incorporating opportunities to move more inside and outside the classroom. TransformUs strategies involve:

- Incorporating movement into class lessons – the delivery of the lesson changes, not the content.
- Making the school environment more supportive of movement throughout the day.

TransformUs is highlighted in the Victorian Department of Education's Active School's toolkit. Professional development opportunities, full lesson plans and supporting resources are made available to participating schools. Please note that teachers can register independently (Principal approval is not required) however we encourage registered teachers to invite their Principal and other school staff to register.

What does my participation involve?

- Two short online surveys (one now and one in 6 months).
- Completion of a one-minute check-in via text approximately three times per term for up to six terms.
- At the end of the project, you may also be contacted to complete an interview about your experiences with TransformUs. The interview will be audio-recorded.

Has this project been approved? Will it be monitored?

Yes, by the Deakin University Human Research Ethics Committee and Education Departments of Victoria, Queensland and South Australia.

Possible benefits and risks

This research will inform further refinement of TransformUs, which is expected to benefit students with a disability, their teachers and the community at a population-level due to the positive outcomes previously demonstrated from this program. There are no foreseeable risks associated with participation in this study. The interview will not ask any personal or sensitive questions.

How privacy and confidentiality will be protected

All participants will be given a study identification number to maintain privacy. All hard copies of personal information (name and contact details) received will be stored in secure cabinets at Deakin University. Any information that is recorded digitally will be saved on secure Deakin servers. Only the research team will have access to this information and no individual data will be identified in the results. Interviews will be transcribed by a professional, university approved transcription service. Deidentified data will be retained and potentially be used for secondary data analysis (for example, data pooling studies). In addition, deidentified data might be made available as a requirement of publication in scientific journals.

Right to withdraw

Your participation in this study is voluntary. Withdrawal is possible at any stage of the research project, and interview data can be withdrawn up to one month following data collection. Your decision will not affect your relationship with Deakin University or the research team in any way.

Any questions about the research?

You can contact the research team on 03 9246 8779 or at transform-all@deakin.edu.au

Complaints

If you have any complaints about any aspect of the project, the way it is being conducted or any questions about your rights as a research participant, then you may contact:

The Human Research Ethics Office, Deakin University, 221 Burwood Highway, Burwood Victoria 3125, Telephone: 9251 7129, research-ethics@deakin.edu.au

Please quote project number [2021-368].

Implementation Trial Consent Form

1. I have read and I understand the attached Plain Language Statement.
2. I have been given a copy of the Plain Language Statement and Consent Form to keep.
3. I freely agree to participate in this project and the evaluation as described in the Plain Language Statement.
4. I understand that I may not be invited to complete an interview.
5. I understand that the interviews with the researchers will be audio recorded.
6. Aggregated results will be used for research purposes and may be reported in scientific and academic journals and conference meetings.
7. The researcher has agreed not to reveal my identity and personal details if information about this project is published or presented in any public form.
8. Deidentified data will be retained and potentially be used for secondary data analysis (for example, data pooling studies). In addition, deidentified data might be made available as a requirement of publication in scientific journals.

My name …………………………………………………….

My role …………………………………………………….

My mobile number: ………………………………………………….

My email address: ………………………………………………….

☐ **By checking this box, I indicate that I have read the Plain Language Statement and consent to take part in this project.**

Plain Language Statement for Principals – Effectiveness Trial

Overview of the project

TransformUs is a learning through movement evidence-based initiative that has been running in Victorian primary schools for over 10 years. Teachers have told us that their students have shown better focus in class and classroom management has improved. We have also observed increases in movement and decreases in sedentary behaviour, as well as health and behavioural benefits in the primary school setting. TransformUs has now been adapted to cater for the additional needs of students with disability, and this project aims to test if the program has similar benefits in these students. This study aims to test the effectiveness of the adapted TransformUs program in relation to health, wellbeing, and behavioural outcomes in students with disability.

The project is being led by Professor Jo Salmon at Deakin University and is funded by the National Health and Medical Research Council.

What is TransformUs?

TransformUs is designed to re-frame the way students learn by incorporating opportunities to move more inside and outside the classroom. TransformUs strategies involve:

- Incorporating movement into class lessons – the delivery of the lesson changes, not the content.
- Making the school environment more supportive of movement throughout the day.

TransformUs is highlighted in the Victorian Department of Education's Active School's toolkit, and was co-designed by teachers and students. Professional development opportunities, full lesson plans and supporting resources are made available to participating schools.

What does my schools' participation involve?

Six Victorian schools will be selected to take part in this trial. We are conducting a waitlist style study design whereby all six schools will be recruited and complete data collection at the same timepoints however 3 schools will be randomly selected to begin the intervention at the beginning of the trial and 3 will be allocated to the control group whereby the intervention would not begin until after follow-up data collection, approximately 6 months later.

<u>Commitment to deliver the initiative in my school</u>

- Provide opportunities for staff to register on the TransformUs website (e.g., during staff meetings).
- A free of charge 2-hour face to face TransformUs Professional Learning (PL) session will be offered to all school staff prior to the start of the intervention. Teacher's participation in this PL is voluntary however we do encourage this to aid with implementation.
- Regularly encourage them to use the strategies in their classrooms and the school grounds.

Recruitment of school staff to be involved in the evaluation

- Distribute the TransformUs information via my school's regular channels of communication.

Provide a contact person for us to liaise with

- Assessments with consenting students will take place at the school and we ask for access to a room to perform these on two occasions approximately 6 months apart. Research staff will work closely with you to minimize any disruption.

What does school staff and student participation involve?

Your school's participation provides an opportunity for us to invite your staff and students to give their consent to participate in the evaluation described below. Parental consent will be required for students to take part.

For consenting school staff

- A free of charge 2-hour face to face TransformUs Professional Learning (PL) session will be offered to all school staff prior to the start of the intervention. Teacher's participation in this PL is voluntary however we do encourage this to aid with implementation.
- Short online survey on two occasions (baseline, 6 months).
- Complete a one-minute check-in via text up to three times per term for during the trial.
    - Sub-sample invited to complete one interview about their experiences with TransformUs, which will be audio-recorded.

For consenting students

Unless stated otherwise assessments will occur two times approximately 6 months apart.

- Wear an activity monitor on their wrist for 8 days.
- Complete a pictorial Physical literacy in Children Questionnaire (PL-C Quest). The PL-C Quest is a pictorial questionnaire that was developed to measure how children perceive their own physical literacy (i.e., the skills, knowledge and motivation to be physically active).
- Cognitive functions: this will be assessed with a computer-based working memory test using iPads/tablets/laptop. The test is simple enough for students with intellectual disability and it will take about 2-4 minutes to complete.
- A sub-sample of students will be invited to participate in short (45-min) group discussions at the end of the study (6 months).

Has this project been approved? Will it be monitored?

Yes, by the Deakin University Human Research Ethics Committee and by the Department of Education and Training of Victoria.

Possible benefits, risks and compensation

This research will provide valuable insight into whether the TransformUs strategies can improve students' activity levels and their enjoyment of school. There are no foreseeable risks associated with participation in this study. If any student expresses concern with any of the assessments on the day, we will not conduct that measurement with that student.

How privacy and confidentiality will be protected

All participants will be given a study identification number to maintain privacy. All hard copies of personal information (name and contact details) received will be stored in secure cabinets at Deakin University. Any information that is recorded digitally will be saved on secure Deakin servers. Only the research team will have access to this information, and no individual data will be identified in the results. Interviews will be transcribed by a professional, university approved transcription service. Deidentified data will be retained and potentially be used for secondary data analysis (for example, data pooling studies). In addition, deidentified data might be made available as a requirement of publication in scientific journals.

Right to withdraw

Your school's participation in this study is voluntary. Withdrawal is possible at any stage of the research project. Your decision will not affect your school's relationship with Deakin University or the research team in any way.

Any questions about the research?

You can contact the research team on 03 9246 8779 or at transform-all@deakin.edu.au.

Complaints

If you have any complaints about any aspect of the project, the way it is being conducted or any questions about your rights as a research participant, then you may contact:
The Human Research Ethics Office, Deakin University, 221 Burwood Highway, Burwood Victoria 3125, Telephone: 9251 7129, research-ethics@deakin.edu.au

Please quote project number [2021-368].

School Consent Form

1. I have read and I understand the attached Plain Language Statement.
2. I have been given a copy of the Plain Language Statement and Consent Form to keep.
3. I give my permission for ***staff/students*** at ***my school*** to participate in this project and the evaluation as described in the Plain Language Statement, if they are willing to participate.
4. I understand I will be required to support the project and disseminate recruitment materials to staff and parents via the school's regular channels of communication.
5. I understand that the interviews with the researchers will be audio recorded.
6. Aggregated results will be used for research purposes and may be reported in scientific and academic journals and conference meetings.
7. The researcher has agreed not to reveal the participants' identities and personal details if information about this project is published or presented in any public form.
8. Deidentified data will be retained and potentially be used for secondary data analysis (for example, data pooling studies). In addition, deidentified data might be made available as a requirement of publication in scientific journals.

School name ……………………………………………………

Name of person giving consent ……………………………………………………

Position/role of person giving consent ……………………………………………………

Contact number: ……………………………………………………

Email address: ……………………………………………………

☐ **By checking this box, I indicate that I have read the Plain Language Statement and consent for my school to take part in this project.**

Plain Language Statement for School Staff - Effectiveness Trial

Overview of the project

TransformUs is a learning through movement evidence-based initiative that has been running in Victorian primary schools for over 10 years. Teachers have told us that their students have shown better focus in class and classroom management has improved. We have also observed increases in movement and decreased sedentary behaviour, as well as health and behavioural benefits in the primary school setting. TransformUs has now been adapted to suit children with special needs, and this project aims to test if the program has similar benefits. This study aims to test the effectiveness of the adapted TransformUs program in relation to health, wellbeing, and behavioural outcomes in students with disability.

This project is being led by Professor Jo Salmon at Deakin University and is funded by the National Health and Medical Research Council.

What is TransformUs?

TransformUs is designed to re-frame the way students learn by incorporating opportunities to move more inside and outside the classroom. TransformUs strategies involve:

- Incorporating movement into class lessons – the delivery of the lesson changes, not the content.
- Making the school environment more supportive of movement throughout the day.

TransformUs is highlighted in the Victorian Department of Education's Active School's toolkit, and was co-designed by school teachers and stakeholders. A 2-hour Face to face TransformUs Professional Learning session will be offered to all teachers as well as full lesson plans and supporting resources are made available to participating schools.
The overarching aim of this project is to explore, adapt and/or further develop the TransformUs program to increase physical activity among primary and secondary school students with disability.

What does my participation involve?

Six Victorian schools will be selected to take part in this Trial. We are conducting a waitlist style study design whereby all six schools will be recruited and complete data collection. However, 3 schools will be selected to begin the intervention at the beginning of the trial and 3 will be randomised to the control group whereby the intervention would not begin until after follow-up data collection, approximately 6 months later.

- Short online survey on two occasions.
- Complete a one-minute check-in via text up to three times per term for the duration of the trial.
- At the end of the project, you may also be contacted to complete an interview about your experiences with TransformUs. The interview will be audio-recorded.

What will consenting students be asked to do?

The assessments below will be conducted at baseline and after 6 months, unless otherwise stated

- Wear an activity monitor on their wrist for 8 days.
- Complete a pictorial Physical literacy in Children Questionnaire (PL-C Quest). The PL-C Quest is a pictorial questionnaire designed to measure how children perceive their own physical literacy (the skills, knowledge and motivation to be physically active).
- Cognitive functions: this will be assessed with a computer-based working memory test using iPads/tablets/laptop. The test is simple enough for students with intellectual disability and it will take about 2-4 minutes to complete.
- A few students will be invited to participate in short (45-min) group discussions at the end of the study (6 months).

Has this project been approved? Will it be monitored?

Yes, by the Deakin University Human Research Ethics Committee and by the Victorian Department of Education.

Possible benefits and risks

This research will provide valuable insight into whether these strategies can improve student's health, wellbeing, and behavioural outcomes. There are no foreseeable risks associated with participation in this study. The interview will not ask any personal or sensitive questions.

How privacy and confidentiality will be protected

All participants will be given a study identification number to maintain privacy. All hard copies of personal information (name and contact details) received will be stored in secure cabinets at Deakin University. Any information that is recorded digitally will be saved on secure Deakin servers. Only the research team will have access to this information, and no individual data will be identified in the results. Interviews will be transcribed by a professional, university approved transcription service. Deidentified data will be retained and potentially be used for secondary data analysis (for example, data pooling studies). In addition, deidentified data might be made available as a requirement of publication in scientific journals.

Right to withdraw

Your participation in this study is voluntary. Withdrawal is possible at any stage of the research project, and interview data can be withdrawn up to one month following data collection. Your decision will not affect your relationship with Deakin University or the research team in any way.

Any questions about the research?

You can contact the research team on 03 9246 8779 or at transform-all@deakin.edu.au

Complaints

If you have any complaints about any aspect of the project, the way it is being conducted or any questions about your rights as a research participant, then you may contact:

The Human Research Ethics Office, Deakin University, 221 Burwood Highway, Burwood Victoria 3125, Telephone: 9251 7129, research-ethics@deakin.edu.au

Please quote project number [2021-368].

School Staff Consent Form

1. I have read and I understand the attached Plain Language Statement.
2. I have been given a copy of the Plain Language Statement and Consent Form to keep.
3. I freely agree to participate in this project and the evaluation as described in the Plain Language Statement.
4. I understand that I may not be invited to complete an interview.
5. I understand that the interviews with the researchers will be audio recorded.
6. Aggregated results will be used for research purposes and may be reported in scientific and academic journals and conference meetings.
7. The researcher has agreed not to reveal my identity and personal details if information about this project is published or presented in any public form.
8. Deidentified data will be retained and potentially be used for secondary data analysis (for example, data pooling studies). In addition, deidentified data might be made available as a requirement of publication in scientific journals.

My name ……………………………………………………

School name …………………………………………………….

My role ……………………………………………………

My mobile number: …………………………………………………..

My email address: …………………………………………………

☐ **By checking this box, I indicate that I have read the Plain Language Statement and consent to take part in this project.**

Plain Language Statement for Students/Parents - Effectiveness Trial

Overview of TransformUs

TransformUs is a learning through movement evidence-based initiative that has been running in Victorian primary schools for over 10 years. Teachers have told us that their students have shown better focus in class and classroom management has improved. We have also observed increases in movement and decreased sedentary behaviour, as well as health and behavioural benefits in the primary school setting. TransformUs has now been adapted to suit children with special needs, and this project aims to test if the program has similar benefits in relation to health, wellbeing, and behavioural outcomes in students with disability.

The program strategies involve:

- Incorporating movement into class lessons – the delivery of the lesson changes, not the content.
- Making the school environment more supportive of movement throughout the day.

We would like to see whether these strategies increase students' physical activity and their enjoyment at school. Your child's school has agreed to take part in TransformUs therefore all students will receive the intervention; however, only students with parental consent will take part in the evaluation outlined below. Your child's school may be selected to begin the intervention at the beginning of the trial or at the end of the Trial (after follow-up data collection, approximately 6 months later).

This project is being led by Professor Jo Salmon at Deakin University and is funded by the National Health and Medical Research Council.

What will my child be asked to do?

Unless stated otherwise assessments will occur two times approximately 6 months apart.

- Wear an activity monitor on their wrist for 8 days.
- Complete a pictorial questionnaire used to assess physical literacy (the Physical literacy in Children Questionnaire). Your child will be presented with 30 questions each containing two pictures with an orange bunny cartoon performing some activities. Each time your child will be asked to pick the picture where they think the cartoon character is being the most like them, if they were in that situation. Once they pick that picture, they will then be asked how much the picture represents them (a bit or a lot). A researcher will be there to help your child to complete the survey. This will take about 8 minutes. See an example below that is not about physical literacy:

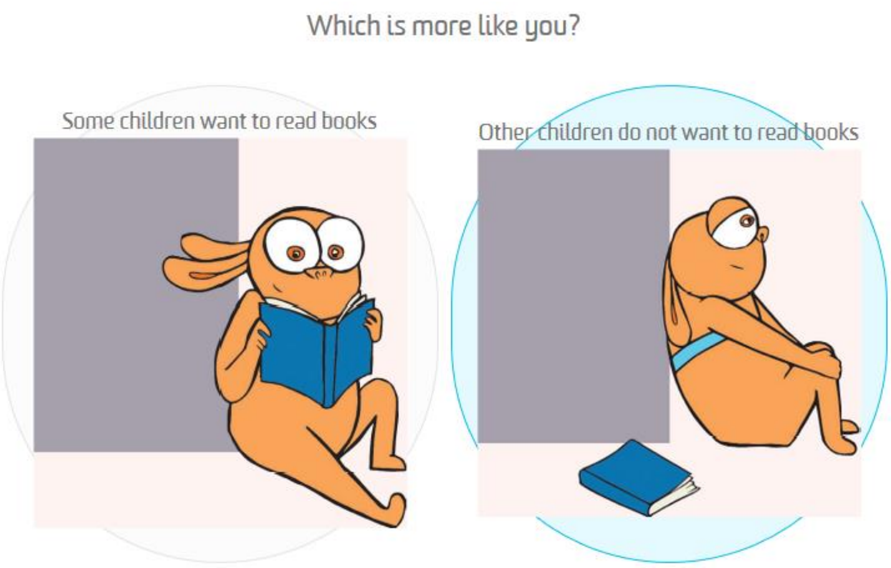

- Cognitive functions will be assessed with a computer-based assessment, where they recall the sequence of some items that appear on the screen by tapping. This is not a clinical test, and it will take about 2-4 minutes to complete.

<u>A sub-sample of students:</u>

- Will be invited to participate in a short (45-min) group discussion at the end of the study (6 months). Students will be randomly selected based on the level of support they require in the classroom.

Has this project been approved? Will it be monitored?

Yes, by the Deakin University Human Research Ethics Committee and by the Victorian Department of Education.

Possible benefits and risks

This research will provide valuable insight into whether these strategies can improve student’s activity levels and behavioural outcomes. There are no foreseeable risks associated with participation in this study. From our experience, students generally find the activities enjoyable. If your child is concerned with any of the assessments on the day, we will not conduct that measurement with them, despite your prior consent to participate.

How privacy and confidentiality will be protected

All participants will be given a study identification number to maintain privacy. All hard copies of personal information (name and contact details) received will be stored in secure cabinets at Deakin University. Any information that is recorded digitally will be saved on secure Deakin servers. Only the research team will have access to this information, and no individual data will be identified in the results. Deidentified data will be retained and potentially be used for secondary data analysis (for example, data pooling studies). In

addition, deidentified data might be made available as a requirement of publication in scientific journals.

Right to withdraw

Your participation in this study is voluntary. Withdrawal is possible at any stage of the research project. Your decision will not affect your relationship with Deakin University or the research team in any way.

Any questions about the research?

You can contact the research team on 03 9246 8779 or at transform-all@deakin.edu.au

Complaints

If you have any complaints about any aspect of the project, the way it is being conducted or any questions about your rights as a research participant, then you may contact:

The Human Research Ethics Office, Deakin University, 221 Burwood Highway, Burwood Victoria 3125, Telephone: 9251 7129, research-ethics@deakin.edu.au

Please quote project number [2021-368].

Student/Parent Consent Form

1. I have read and I understand the attached Plain Language Statement.
2. I have been given a copy of the Plain Language Statement and Consent Form to keep.
3. I freely agree to participate in this project and the evaluation as described in the Plain Language Statement.
4. I understand that my child may not be invited to participate in a discussion group.
5. Aggregated results will be used for research purposes and may be reported in scientific and academic journals and conference meetings.
6. An aggregated summary of the results will be sent to the participating schools, teachers and parents.
7. The researcher has agreed not to reveal the participants' identities and personal details if information about this project is published or presented in any public form.
8. Deidentified data will be retained and potentially be used for secondary data analysis (for example, data pooling studies). In addition, deidentified data might be made available as a requirement of publication in scientific journals.

Parent/guardian consent

☐ **By checking this box I, the parent/guardian, indicate that I have read the Plain Language Statement and consent for my child to take part in the study.**

Optional consent – information about future research

☐ I do	☐ I do not	**Consent to being sent information about future research**

Student name: ____

Student date of birth: ___ ___ / ___ ___ / ___ ___ ___ ___

Student year level: _______________

School name: ____

Parent/ guardian name: ____

Parent/ guardian mobile: ____

Parent/guardian email address: ____
