# Supplementary material for: School-based strategies to increase physical activity and reduce sedentary behaviour in students with disability: protocol of the TransformUs All Abilities hybrid type II implementation-effectiveness trial
Source: BMJ Open. 2025 Nov 9;15(11):e105311. doi: 10.1136/bmjopen-2025-105311 (PMC12598974; doi:10.1136/bmjopen-2025-105311)
Supplement: Supplementary file 3 [file bmjopen-2025-105311.reviewer_comments.pdf]

PEER REVIEW HISTORY

BMJ Open publishes all reviews undertaken for accepted manuscripts. Reviewers are asked to complete a checklist review form and are provided with free text boxes to elaborate on their assessment. These free text comments are reproduced below.

ARTICLE DETAILS

Title (Provisional)

School-based strategies to increase physical activity and reduce sedentary behaviour in students with disability: protocol of the TransformUs All Abilities hybrid type II implementation-effectiveness trial

Authors

Mazzoli, Emiliano; Contardo Ayala, , Ana María; Koorts, Harriet; Timperio, Anna; Lander, Natalie; Lubans, David Revalds; Ridgers, Nicola D.; Anderson, Kate Louise McCann; Cairney, John; Barnett, Lisa Michele; Salmon, Jo

---

VERSION 1 - REVIEW

**Reviewer**	**1**
**Name**	**Khudair, Mohammed**
**Affiliation**	**Northumbria University - City Campus, Sport, Exercise and Rehabilitation**
**Date**	**30-Jun-2025**
**COI**	**None**

The current manuscript describes a protocol of the TransformUs All Abilities trial which aims to increase physical activity/reduce sedentary behaviour in children and adolescents with disabilities. This is an adaptation of the TransformUs trial to target children and adolescents with disabilities. The original trial was based on teaching strategies and environmental adaptation to facilitate physical activity (e.g., equipment provision) and limit sedentary behaviour (e.g., active classrooms) and has shown promising results (particularly relating to reductions in SB). The current version "All Abilities" provides additional adaptation to intervention activities to support the abilities and enjoyment of children and adolescents with disabilities. The methodology is strong and follows the previous version of the trial. I have some minor concerns, relating to the application of the trial in people with disability, that I think can be addressed easily.

1. Considering the adaptation of the trial to a population of children and adolescents with disabilities, consider providing briefly some insight into the previous trial, pros/cons/results

and why it is worth adapting. That is, what about the TransformUs intervention made it worthy of being adapted? Consider elaborating on this point.

2. The general term disability is used throughout the manuscript. The authors note that there is a wide variation in limitations faced by children and adolescents with disabilities, mostly depending on the classification of disability. Therefore, “children and adolescents with disabilities” is a broad population with a variety of considerations. How is this variation expected to influence the success, implementation and reception of the trial and what has been considered in the decision and process to adapt the trial? Consider elaborating on this in the introduction.

3. P3, L55-60: It is not entirely clear from the abstract whether this protocol describes two separate trials or a single trial with two objectives of analysis.

4. P6, L138-141: Considering that this paper describes the protocol of an implementation-effectiveness trial aiming to increase PA/reduce SB, how does it address the health-related impact?

5. P11-12, L265-267: The authors mention that the effectiveness trial will be involve schools with children with intellectual disability. The authors also acknowledge the variation among disability classifications and the need to adapt the trial/instructions accordingly (P10, L 229-238). Please, consider providing more detail on why there will be focus on this particular disability and what it means for generalisability “All Abilities”. Also, is this a strength or a limitation?

VERSION 1 - AUTHOR RESPONSE

Reviewer: 1<br>Dr. Mohammed Khudair, Northumbria University - City Campus, Cardiff University	Response	Changes to the manuscript<br>(i.e., Main Document – marked copy)
The current manuscript describes a protocol of the TransformUs All Abilities trial which aims to increase physical activity/reduce sedentary behaviour in children and adolescents with disabilities. This is an adaptation of the TransformUs trial to target children and adolescents with disabilities. The original trial was based on	We appreciate the positive comments and the careful review which have helped us to strengthen our manuscript.	n/a

teaching strategies and environmental adaptation to facilitate physical activity (e.g., equipment provision) and limit sedentary behaviour (e.g., active classrooms) and has shown promising results (particularly relating to reductions in SB). The current version “All Abilities” provides additional adaptation to intervention activities to support the abilities and enjoyment of children and adolescents with disabilities. The methodology is strong and follows the previous version of the trial. I have some minor concerns, relating to the application of the trial in people with disability, that I think can be addressed easily.		
1. Considering the adaptation of the trial to a population of children and adolescents with disabilities, consider providing briefly some insight into the previous trial, pros/cons/results and why it is worth adapting. That is, what about the TransformUs intervention made it worthy of being adapted? Consider elaborating on this point.	We thank the reviewer for this comment, which highlights the need to clarify that the trials conducted so far to assess efficacy, effectiveness, implementation, and scale-up of TransformUs did not target students with disability. Whilst we previously provided an overview of the TransformUs program component and the available promising evidence on how the program can support healthy behaviours in children (Page 8–9, Lines 179–199, References 42-49), we have now made it	Page 8, Lines 179–181: *“TransformUs[42] is an efficacious whole-of-school program delivered in the primary school setting that improved ~~children’s~~ health, successfully reduced sitting, and increased physical activity at school and home in children without disability.[43-46]”*<br><br>Page 9, Lines 194–198: *“Recognising this gap, and in response to direct demand from educators and schools serving students with disability, the program has recently been adapted (i.e.,*

	clearer that those trials did not target students with disability. As the currently available evidence cannot be generalized to students with disability, empirically testing the effectiveness and implementation of TransformUs in this cohort of students is necessary and important for equity.<br><br>Furthermore, TransformUs was not originally designed considering the additional needs of children and adolescents with disability. Recognising this gap, and in response to direct demand from educators and schools serving students with additional needs, the TransformUs All Abilities trial was developed.<br><br>As described in Pages 12, the adaptations were co-designed with parents and key organisations from education and disability, to ensure that the strategies and resources offered could facilitate more inclusive movement-based practices in schools. These modifications aim to support diverse abilities and enhance enjoyment and engagement, making the program accessible and meaningful for students with a wide range of functional and cognitive profiles. We have also clarified this point.	*TransformUs All Abilities) to ensure that the additional ~~for the specific~~ needs of primary and secondary students with disability, in ~~primary and secondary (~~mainstream or special~~)~~ schools, are considered~~(i.e., TransformUs All Abilities)~~.”*<br><br>Page 11, Lines 260–263: *“The adaptations are designed to support students with a wide range of functional and cognitive abilities, aiming to enhance enjoyment and engagement, and ensure the program is both accessible and meaningful for all students”*

2. The general term disability is used throughout the manuscript. The authors note that there is a wide variation in limitations faced by children and adolescents with disabilities, mostly depending on the classification of disability. Therefore, "children and adolescents with disabilities" is a broad population with a variety of considerations. How is this variation expected to influence the success, implementation and reception of the trial and what has been considered in the decision and process to adapt the trial? Consider elaborating on this in the introduction.	Thanks for this point on the heterogeneity of disability. In Pages 10 and 11 we acknowledged the challenge of targeting different disability and discussed the implications of this in relation to adapting TransformUs. We described how this diversity informed our adaptation process, including flexible implementation strategies and tailored support mechanisms. Based on the social model of disability, we acknowledge that disability results from the environmental and societal barriers that individuals encounter.<br><br>For TransformUs All Abilities we made a deliberate decision focusing on the social model of disability (rather than the medical model); for this reason, the proposed classification we offer broader categories of difficulties within certain domains (i.e., physical, sensory, cognitive, and/or social-emotional). This approach is practical and more universal – in the sense that can apply to children diagnosed with disability as well as those who may have temporary difficulties with certain domains – than the one offered by medical/diagnostic categorisations.	Pages 10–11, Lines 230–251:<br>*"The adaptation of TransformUs followed a strengths-based approach inspired by principles of inclusive education. Targeting a wide range of disabilities, grouping them appropriately was an initial challenge, as people of disability may present highly heterogeneous physical, cognitive, sensory, and behavioural needs. Whilst frameworks like the International Classification of Functioning, Disability and Health (ICF)[52] and the Diagnostic and Statistical Manual of Mental Disorders (DSM)[53] offer detailed categorisations, these are complex and not practical for group-based interventions. In designing TransformUs All Abilities, we adopted a broad and inclusive classification of disability aligned with the social model of disability, which emphasises the role of societal and environmental barriers in limiting participation, rather than focusing solely on individual impairments. Therefore, we adopted a broader approach that acknowledges the diversity of experiences and needs among children and adolescents with disabilities. Rather than relying on narrow diagnostic categories, we*

	The adaptations were developed to address the additional needs of students facing difficulties with one or more of the domains described above. While there is wide variation in disability types, in the co-design approach and the pilot work (References 36 and 38) taken to adapt resources, we considered disability as comprehensively as possible aiming to provide solutions that could be flexibly tailored to suit the specific needs of each student.<br><br>We have now added a few sentences to expand these ideas in the manuscript.	*aligned our classification with the Nationally Consistent Collection of Data on School Students with Disability (NCCD),[54] which identifies four broad categories of disability: sensory, physical, cognitive, and social-emotional. This framework reflects the social model of disability, recognising that difficulties within each of these broad domains often arise from the interaction between individuals and their environments, rather than from impairments alone. By framing disability in this way, TransformUs All Abilities aims to promote universal design principles and inclusive practices that support participation for all students, regardless of diagnosis or classification. This simple and universal framework focuses on students' functional needs and provides strategies applicable to all students, not just those with disabilities."*
3. P3, L55-60: It is not entirely clear from the abstract whether this protocol describes two separate trials or a single trial with two objectives of analysis.	The protocol describes a hybrid trial with two objectives (one focusing on intervention effectiveness and the other related to implementation). We have now clarified this in the abstract.	Page 3, Line 56:<br>*"This article describes ~~two~~ the hybrid implementation-effectiveness trial~~s~~ that will be used for this evaluation."*
4. P6, L138-141: Considering that this paper describes the	Physical activity and sedentary behaviour are related two types of	n/a

protocol of an implementation-effectiveness trial aiming to increase PA/reduce SB, how does it address the health-related impact?	behaviours that are strongly associated to various health outcomes in young people. Besides these outcomes, the trials will also explore intervention impact on students' cognitive functions (i.e., working memory, which could be considered a health outcome) and physical literacy. Whilst all these outcomes relate to health, the trial does not aim to directly investigate the impact of the program on physical/mental health outcomes.	
5. P11-12, L265-267: The authors mention that the effectiveness trial will be involve schools with children with intellectual disability. The authors also acknowledge the variation among disability classifications and the need to adapt the trial/instructions accordingly (P10, L 229-238). Please, consider providing more detail on why there will be focus on this particular disability and what it means for generalisability "All Abilities". Also, is this a strength or a limitation?	We appreciate this is a valid point. To ensure comparability of findings across different children involved in the effectiveness testing, we were compelled to restrict our study to a specific group. Given the many barriers to participation encountered by individuals with intellectual disability, we deemed this group be a good sample to focus on. Given the broad range of disability types that exist, investigating effectiveness in a diverse sample representative of all disabilities would require a substantial sample size to ensure. However, we acknowledge that the results obtained from this group may not be generalisable to children with different disability types and have added a	Page 5, Lines 111–115:<br><br>*"The trial's six-month duration provides initial insights into program implementation and short-term outcomes, though it may limit understanding of real-world impact, and while implementation includes all school types and has the potential to benefit students with a wide range of (dis-)abilities, effectiveness mainly reflects outcomes for students with intellectual disability and may not be generalisable to other disability types."*

	clarification of this in our limitation section.<br><br>The implementation component is open to every school type and can potentially reach students with various types of disabilities.	

VERSION 2 - REVIEW

**Reviewer**	**1**
**Name**	**Khudair, Mohammed**
**Affiliation**	**Northumbria University - City Campus, Sport, Exercise and Rehabilitation**
**Date**	**16-Sep-2025**
**COI**	

I would like to commend the authors on their stellar work. The authors have addressed all my concerns and I think that the current version of the manuscript clearly outlines the protocol for the intended trial "TransformUs All Abilities". The authors have also laid out potential limitations that indicate forethought to potential issues that may arise. This is helped by the fact that the "TransformUs" trial has previously been tested in children without disabilities, giving insight into implementation.

VERSION 2 - AUTHOR RESPONSE

Reviewer: 1<br>Dr. Mohammed Khudair, Northumbria University - City Campus, Cardiff University	Response	Changes to the manuscript (i.e., Main Document – marked copy)
I would like to commend the authors on their stellar work. The authors have addressed all my concerns and I think that the current version of the manuscript clearly outlines the protocol for the intended	We appreciate the positive comments and the careful review which have helped us to strengthen our manuscript.	n/a

trial "TransformUs All Abilities". The authors have also laid out potential limitations that indicate forethought to potential issues that may arise. This is helped by the fact that the "TransformUs" trial has previously been tested in children without disabilities, giving insight into implementation.		
